# Bacterial Argonaute Proteins Aid Cell Division in the Presence of Topoisomerase Inhibitors in Escherichia coli

**DOI:** 10.1128/spectrum.04146-22

**Published:** 2023-04-27

**Authors:** Anna Olina, Aleksei Agapov, Denis Yudin, Dmitry Sutormin, Alina Galivondzhyan, Anton Kuzmenko, Konstantin Severinov, Alexei A. Aravin, Andrey Kulbachinskiy

**Affiliations:** a Institute of Molecular Genetics, National Research Center “Kurchatov Institute”, Moscow, Russia; b Skolkovo Institute of Science and Technology, Moscow, Russia; c Division of Biology and Biological Engineering, California Institute of Technology, Pasadena, California, USA; d Waksman University for Microbiology, Rutgers, New Jersey, USA; e Institute of Gene Biology, Russian Academy of Sciences, Moscow, Russia; University of Manitoba

**Keywords:** prokaryotic Argonautes, gyrase, DNA replication, *ter* sites, cell division, DNA gyrase, antibiotic resistance

## Abstract

Prokaryotic Argonaute (pAgo) proteins are guide-dependent nucleases that function in host defense against invaders. Recently, it was shown that TtAgo from Thermus thermophilus also participates in the completion of DNA replication by decatenating chromosomal DNA. Here, we show that two pAgos from cyanobacteria Synechococcus elongatus (SeAgo) and Limnothrix rosea (LrAgo) are active in heterologous Escherichia coli and aid cell division in the presence of the gyrase inhibitor ciprofloxacin, depending on the host double-strand break repair machinery. Both pAgos are preferentially loaded with small guide DNAs (smDNAs) derived from the sites of replication termination. Ciprofloxacin increases the amounts of smDNAs from the termination region and from the sites of genomic DNA cleavage by gyrase, suggesting that smDNA biogenesis depends on DNA replication and is stimulated by gyrase inhibition. Ciprofloxacin enhances asymmetry in the distribution of smDNAs around Chi sites, indicating that it induces double-strand breaks that serve as a source of smDNA during their processing by RecBCD. While active in E. coli, SeAgo does not protect its native host *S. elongatus* from ciprofloxacin. These results suggest that pAgo nucleases may help to complete replication of chromosomal DNA by promoting chromosome decatenation or participating in the processing of gyrase cleavage sites, and may switch their functional activities depending on the host species.

**IMPORTANCE** Prokaryotic Argonautes (pAgos) are programmable nucleases with incompletely understood functions *in vivo*. In contrast to eukaryotic Argonautes, most studied pAgos recognize DNA targets. Recent studies suggested that pAgos can protect bacteria from invader DNA and counteract phage infection and may also have other functions including possible roles in DNA replication, repair, and gene regulation. Here, we have demonstrated that two cyanobacterial pAgos, SeAgo and LrAgo, can assist DNA replication and facilitate cell division in the presence of topoisomerase inhibitors in Escherichia coli. They are specifically loaded with small guide DNAs from the region of replication termination and protect the cells from the action of the gyrase inhibitor ciprofloxacin, suggesting that they help to complete DNA replication and/or repair gyrase-induced breaks. The results show that pAgo proteins may serve as a backup to topoisomerases under conditions unfavorable for DNA replication and may modulate the resistance of host bacterial strains to antibiotics.

## INTRODUCTION

Argonaute (Ago) proteins are an evolutionary conserved family of programmable nucleases that are found in all three domains of life (1–4). Eukaryotic Argonautes (eAgos) participate in RNA interference and use small RNA guides to recognize RNA targets (5–8). This is followed by target RNA cleavage through an intrinsic endonucleolytic activity of eAgo or by recruitment of accessory factors, resulting in posttranscriptional or transcriptional gene silencing (9–12). When first identified in bacteria and archaea, prokaryotic Argonautes (pAgos) served as models to study the structure and biochemical properties of Ago proteins (3, 13–19), but their functional activities in host species remained unknown. Full-length eAgos and pAgos contain four main domains, N-terminal, PAZ (Piwi/Argonaute/Zwille), MID (middle), and PIWI (P-element Induced WImpy testis), responsible for interactions with the guide and target molecules and for target cleavage, with a catalytic tetrad located in the PIWI domain. However, phylogenetic analysis demonstrated that pAgos are much more diverse than eAgos and only a smaller part of them is catalytically active, while others contain substitutions of the catalytic residues and may include only MID and PIWI domains (1, 2, 4, 20, 21). This analysis also revealed widespread horizontal transfer of pAgos among bacterial and archaeal species, suggesting that they can function in various genetic contexts and environments.

*In vitro* studies of pAgo proteins demonstrated that their primary target is DNA, except for a small group of pAgos that can target RNA, discovered recently (22, 23). Recognition of DNA targets by pAgos can be guided by small DNAs, as observed for most studied pAgos, or by small RNAs (24–33). These findings were corroborated by *in vivo* analysis of several DNA-targeting pAgo proteins in bacterial cells, which revealed that pAgos preferentially recognize foreign DNA, such as plasmids, mobile elements, and phages. In particular, it was shown that RsAgo from Rhodobacter sphaeroides, TtAgo from Thermus thermophilus, PfAgo from Pyrococcus furiosus, MjAgo from Methanocaldococcus jannaschii, and CbAgo from Clostridium butyricum decrease plasmid DNA content and transformation efficiency, and CbAgo counteracts phage infection (25, 29, 31–34). Accordingly, these pAgos are actively loaded with guide molecules corresponding to plasmid or phage sequences during their expression in Escherichia coli (29, 32, 34). As demonstrated for CbAgo, generation of small guide DNAs from foreign genetic elements depends on both the catalytic activity of pAgo and the action of cellular nucleases (34). These studies suggested that catalytically active pAgos perform targeted degradation of foreign DNA, even if they are expressed in heterologous cells.

Recent studies suggested that elimination of invader DNA may not be the sole function of pAgos. In particular, TtAgo was shown to increase the resistance of its host thermophilic bacterium T. thermophilus to ciprofloxacin (Cfx), an inhibitor of DNA gyrase that impairs DNA replication and prevents normal cell division (35). TtAgo was shown to target the region of replication termination and participate in decatenation of chromosomal DNA, thus helping to complete DNA replication when the gyrase function is inhibited (35). However, it remained unknown whether this function in cell division might be conserved among other DNA-targeting pAgos.

Here, we have analyzed two pAgo proteins from mesophilic cyanobacteria, SeAgo from Synechococcus elongatus and LrAgo from Limnothrix rosea. SeAgo is more closely related to TtAgo (35.7% identity in the MID and PIWI domains), while LrAgo is more distant from it on the phylogenetic tree (25.8% identity) (20). Both SeAgo and LrAgo are DNA-guided DNA nucleases that can perform precise cleavage of target DNA *in vitro* at moderate temperatures, suggesting that they may be active in other mesophilic bacteria (27, 28). SeAgo was previously shown to interact with small guide DNAs in its native host *S. elongatus* but without obvious target specificity (28). We have found that, when expressed in a heterologous E. coli host, both SeAgo and LrAgo are loaded with small DNAs corresponding to the sites of replication termination. Furthermore, both SeAgo and LrAgo increase E. coli resistance to the gyrase inhibitor ciprofloxacin, suggesting that targeting of termination sites by pAgos may aid DNA replication in various prokaryotic species.

## RESULTS

### Cyanobacterial pAgos rescue E. coli growth in the presence of topoisomerase inhibitors.

**FIG 1:**
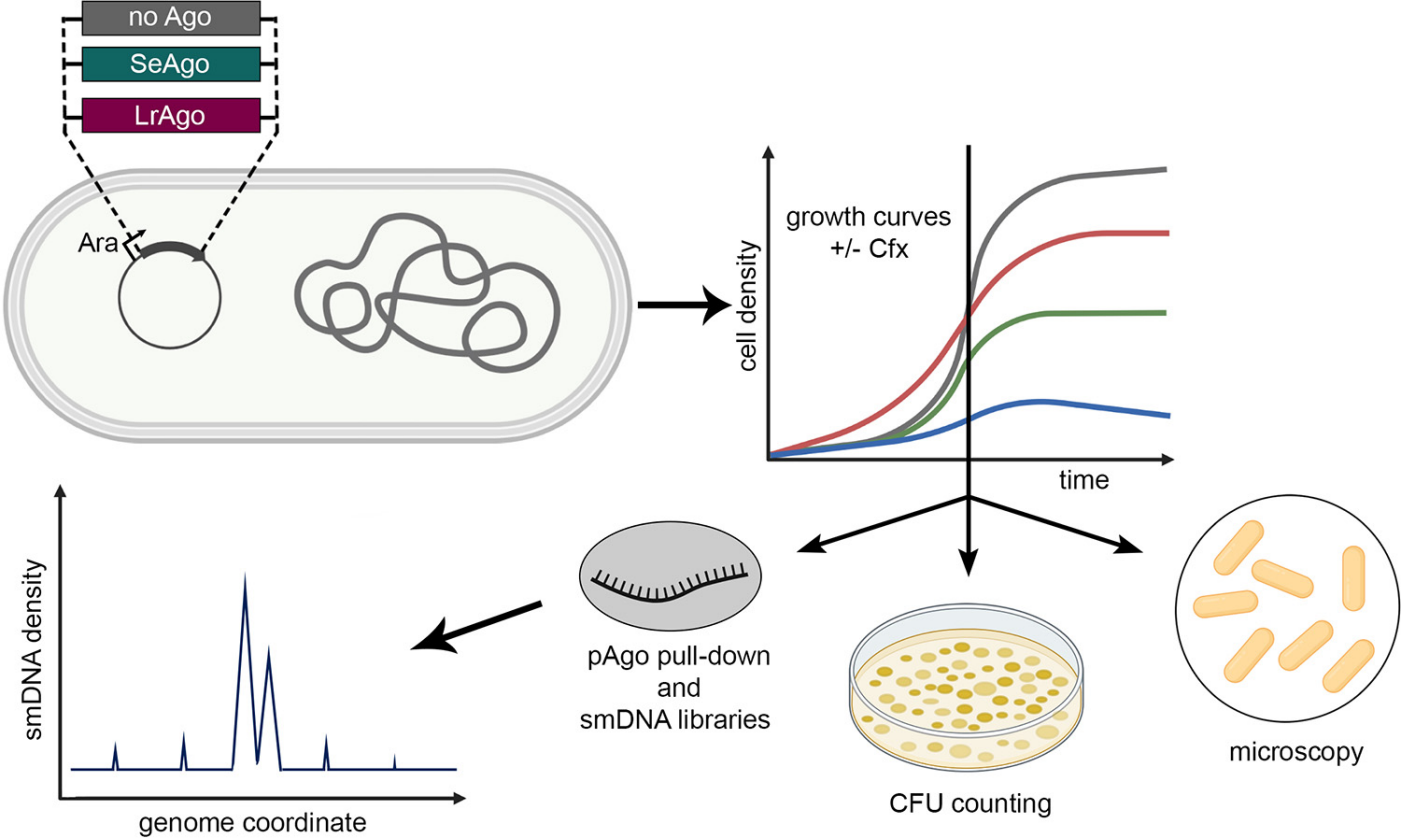
Analysis of pAgo functions in E. coli. E. coli strains expressing plasmid-encoded SeAgo or LrAgo or containing a control empty plasmid were grown in the absence or in the presence of ciprofloxacin (Cfx), followed by CFU counting, cell microscopy, and analysis of pAgo-associated smDNAs.

To explore whether SeAgo and LrAgo can affect DNA replication and cell division, we expressed them in the heterologous E. coli system. The SeAgo and LrAgo genes were cloned under the control of an arabinose-inducible promoter in pBAD-based vectors (Fig. 1). Western blots confirmed that both proteins were expressed after the addition of arabinose (Ara) (see Fig. S1A in the supplemental material). We then studied their effects on cell growth and resistance to gyrase inhibition and further analyzed their DNA specificity *in vivo* (Fig. 1).

**FIG 2:**
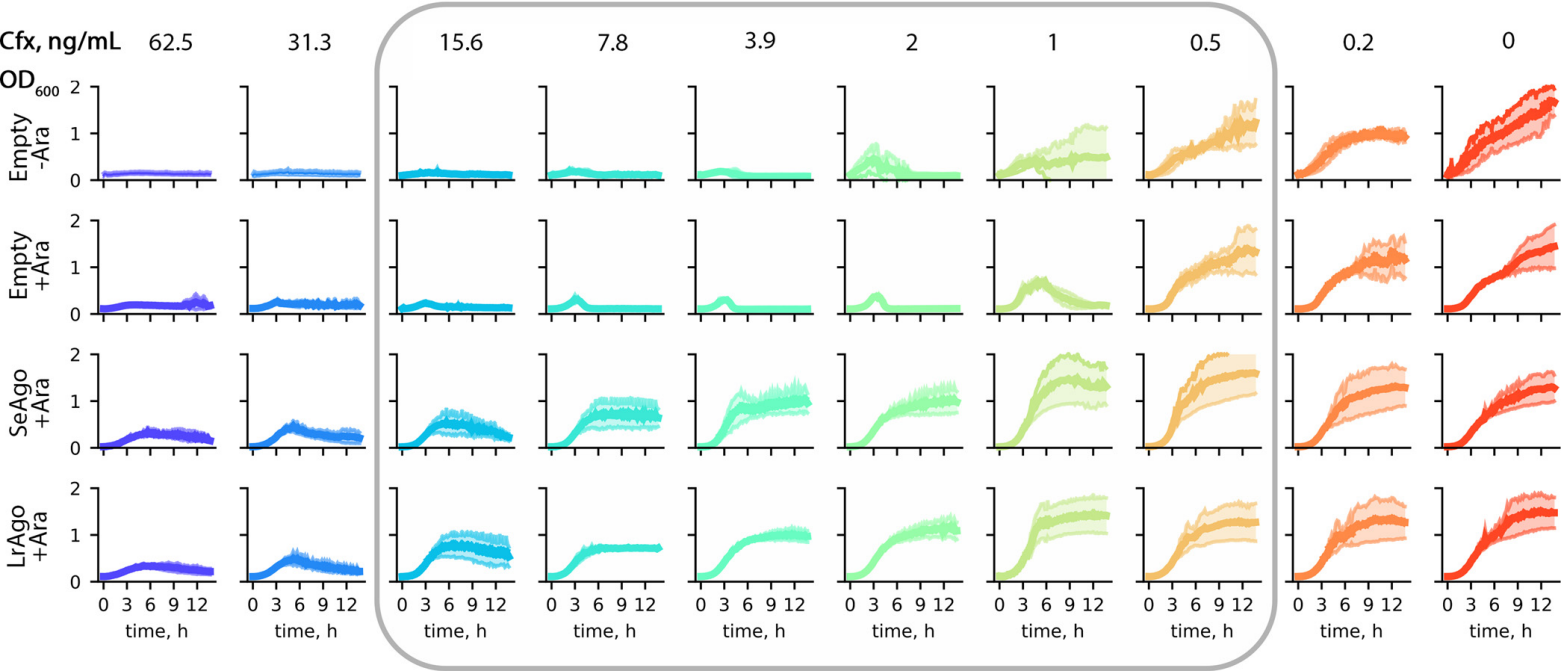
Growth of E. coli strains lacking or expressing pAgos at different concentrations of Cfx. E. coli transformed with an empty pBAD plasmid or pBAD encoding SeAgo or LrAgo were grown at 30°C in a plate reader with indicated concentrations of Cfx (0 to 62.5 ng/mL) and Ara to induce expression of pAgos, and OD_600_ was monitored over time. E. coli transformed with empty pBAD and grown without Ara was used as a control. Means from four biological replicates are shown; shades represent 0.95 confidence intervals of the mean. Gray rectangle highlights the range of Cfx concentrations (0.5 to 15.6 ng/mL), the inhibitory effects of which are suppressed by pAgos expression.

We first determined the range of sublethal concentrations of ciprofloxacin (Cfx) in the absence of pAgo proteins by measuring the kinetics of cell growth (optical density at 600 nm [OD_600_] curves) of an E. coli strain containing an empty expression plasmid, either in the absence or in the presence of Ara. In both cases, cell growth was partially inhibited starting from 0.2 to 0.5 ng/mL of Cfx and was completely inhibited at concentrations higher than 2 ng/mL, which was defined as the MIC of Cfx (Fig. 2, top two rows). We then tested the effects of the pAgo proteins on cell growth. LrAgo and SeAgo did not affect the growth kinetics in the absence of Cfx; however, both proteins protected the cells from the sublethal and lethal concentrations of Cfx (0.5 to 15.6 ng/mL or 0.25 to 8 MIC) (Fig. 2, bottom two rows). Comparable effects were observed at different concentrations of the Ara inducer (0.01% and 0.1%) (Fig. S1B). We therefore used milder expression conditions (with lower Ara) in all subsequent experiments. Even at high Cfx concentrations (31 to 62 ng/mL or 16 to 32 MIC), pAgos partially restored cell growth, which was completely absent in the control conditions (Fig. 2). These experiments suggested that both pAgos can help the cells overcome the inhibitory effects of Cfx on DNA replication.

**FIG 3:**
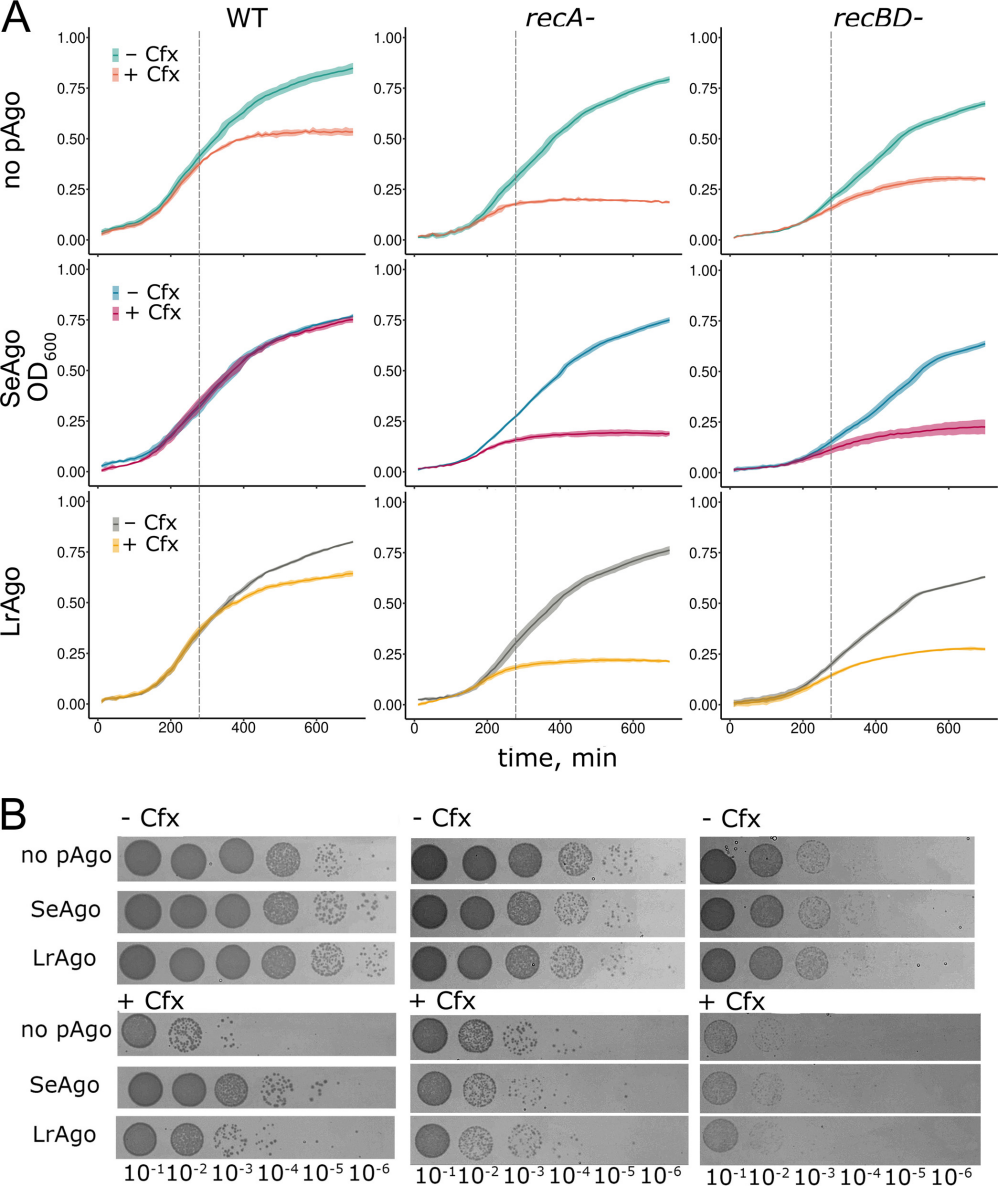
Comparison of the effects of pAgos on the viability of wild-type and *rec*-minus E. coli strains grown in the absence and in the presence of Cfx. (A) Growth kinetics of the wild-type and *recA*-minus and *recBD*-minus strains, measured in a plate reader. Cfx was added to 0.5 ng/mL when indicated. Means and standard deviations from three biological replicates are shown. (B) Comparison of the number of viable cells (CFU) in the wild-type and mutant E. coli strains lacking or expressing pAgos in the absence and in the presence of Cfx. The samples were taken from E. coli cultures grown for 4.5 h in the absence or in the presence of Cfx (indicated with dashed lines in panel A), and CFU numbers were determined by their serial plating without Cfx. Representative LB plates from one of biological replicates are shown for E. coli strains grown in the absence (top) or in the presence (bottom) of Cfx (see Fig. S2 in the supplemental material for CFU numbers from the three biological replicates).

Previous experiments with TtAgo suggested that it helps to decatenate chromosomes by directly introducing double-strand breaks in the genomic DNA of T. thermophilus or by assisting their processing by the DNA repair machinery (35). If SeAgo and LrAgo acted by a similar mechanism, double-strand breaks generated during this process should subsequently be repaired by homologous recombination, depending on the RecA protein and the RecBCD helicase-nuclease involved in double-strand break processing (36–38). To test whether this was the case, we compared the effects of Cfx and pAgos in wild-type and mutant E. coli strains with deletions of *recA* as well as *recB* and *recD*. As expected, Cfx had stronger effects on the growth of the mutant strains in comparison with wild-type cells (Fig. 3A). Notably, SeAgo and LrAgo did not stimulate the growth of *recA*-minus and *recBD*-minus strains in the presence of Cfx (Fig. 3A), suggesting that the function of the pAgo proteins depends on the homologous recombination machinery.

**FIG 4:**
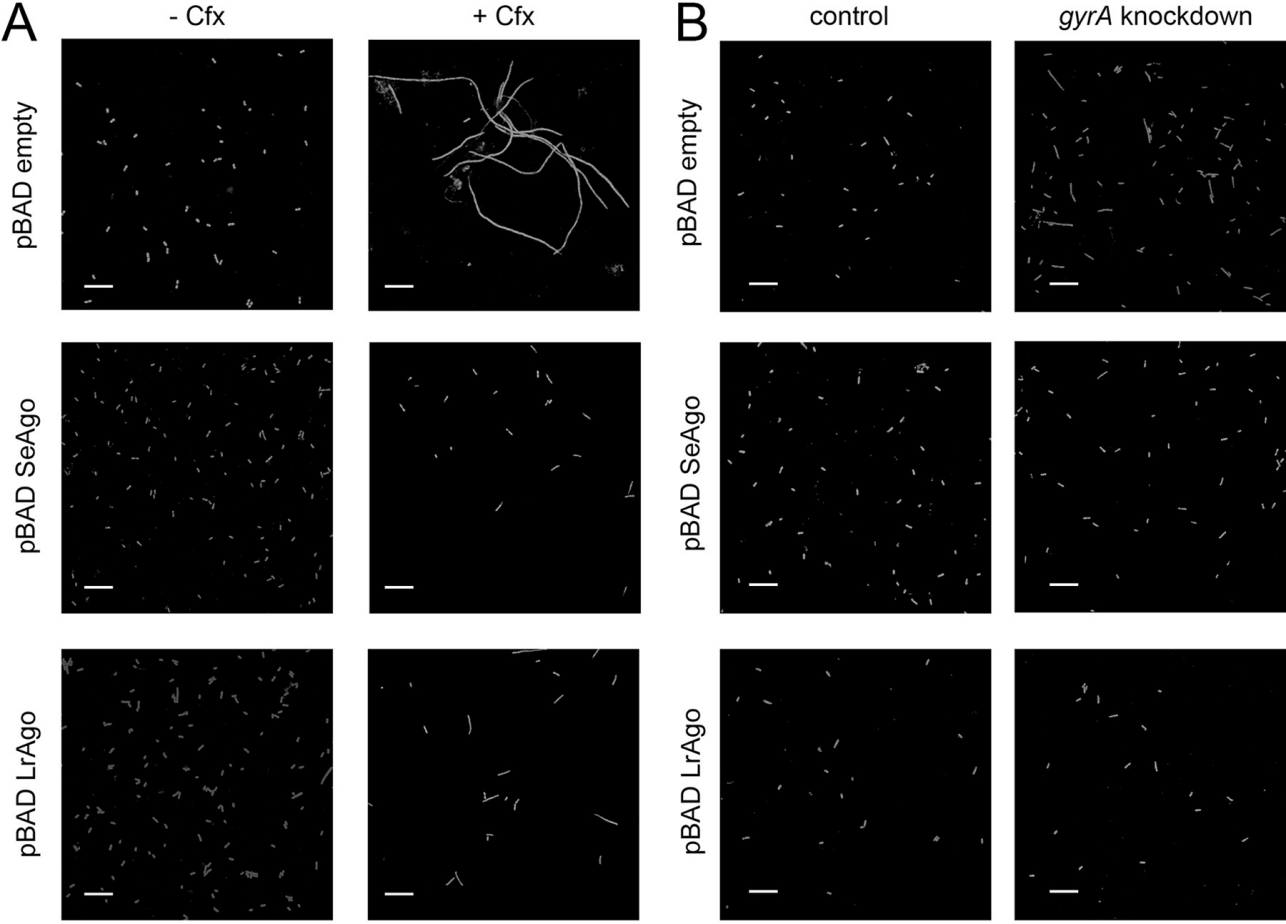
Effects of pAgo expression and gyrase inhibition on E. coli cell morphology. (A) E. coli cells lacking or containing pAgos were grown in the absence (left) or in the presence (right) of Cfx. The samples were taken at 4.5 h from the cultures shown in Fig. 3. (B) Effects of dCas9 gyrase (*gyrA*) knockdown on cell morphology. Fluorescence microscopy after acridine orange staining. The scale bar is 10 μm. See Fig. S3 in the supplemental material for additional fields of view.

The observed effects of Cfx on the density of cell cultures may not directly correspond to changes in the number of viable bacteria because Cfx induces formation of multinucleate cell filaments that increase culture density but do not divide properly (39) (see Discussion). We therefore directly compared the number of CFU and analyzed cell morphology in bacterial cultures grown in the absence and in the presence of Cfx. After 4.5 h of growth, when the effects of Cfx just became visible on the growth curves obtained by optical density measurements (Fig. 3A), Cfx dramatically decreased CFU numbers in the wild-type E. coli strain (20- to 360-fold in three biological replicates) (Fig. 3B; see also Fig. S2 in the supplemental material). Similar differences in CFU numbers were observed for the *rec*-minus strains (with lower numbers of viable cells in the *recBD* strain even in the absence of Cfx) (Fig. 3B). Microscopy analysis confirmed that inhibition of cell division by Cfx caused formation of long filamentous cells containing unsegregated DNA (Fig. 4A, top; see also Fig. S3 in the supplemental material).

We then analyzed the effects of pAgo expression on the number of viable bacteria. In the absence of Cfx, CFU numbers were similar in control and pAgo-expressing strains. In contrast, in the presence of Cfx, CFU numbers were strongly increased in the strains expressing pAgos (Fig. 3B; see also Fig. S2). This effect was especially prominent in the case of SeAgo; for this strain, Cfx decreased cell numbers only 3- to 16-fold in comparison with an up to 360-fold reduction observed in the control strain. Microscopy analysis showed that expression of both SeAgo and LrAgo resulted in disappearance of long filaments induced by Cfx and increased the number of normal cells and shorter filaments. This effect was especially pronounced for SeAgo, while elongated cells were still observed in the case of LrAgo (Fig. 4A, middle and bottom; see also Fig. S3).

Since Cfx primarily targets gyrase, we analyzed the effects of gyrase knockdown on cell morphology in control cells and upon pAgos expression. To silence gyrase expression, catalytically inactive dCas9 and single guide RNA (sgRNA) corresponding to the beginning of the coding region of the *gyrA* gene were expressed in the E. coli strains lacking or containing pAgos. Control experiments demonstrated that only a low level of gyrase knockdown could be achieved by this approach (10 to 20% decrease in the mRNA levels measured by quantitative PCR). However, this was sufficient to observe formation of short cell filaments by microscopy (Fig. 4B, top). Similarly to the experiments with Cfx, these filaments disappeared in the presence of pAgos (Fig. 4B, middle and bottom). Together, these results suggest that pAgos can aid cell division in E. coli cells when gyrase is inhibited by either Cfx or transcriptional knockdown, indicating that they may help to complete DNA replication impaired by topoisomerase deficiency.

### pAgo-bound smDNAs are enriched in the termination region of the chromosome and are generated with participation of RecBCD.

Previous analysis of small guide DNAs (smDNAs) associated with TtAgo in T. thermophilus demonstrated that they are highly enriched around the region of replication termination. This led to the suggestion that TtAgo may facilitate decatenation of chromosomal DNA by targeting the *ter* region of the chromosome (35). To explore whether SeAgo and LrAgo have preference for specific genomic regions or sequence motifs, we checked whether they were loaded with smDNAs during their expression in the heterologous E. coli host. Electrophoretic analysis of nucleic acids copurified with SeAgo and LrAgo revealed that both pAgos were associated with ~14- to 19-nucleotide (nt) smDNAs (see Fig. S4 in the supplemental material). We sequenced libraries of pAgo-associated smDNAs obtained from late logarithmic or stationary bacterial cultures (for 5.5- and 12.5-h time points) (Fig. S4) in the absence or in the presence of Cfx and analyzed the distribution of smDNAs along the chromosomal and plasmid DNAs.

**FIG 5:**
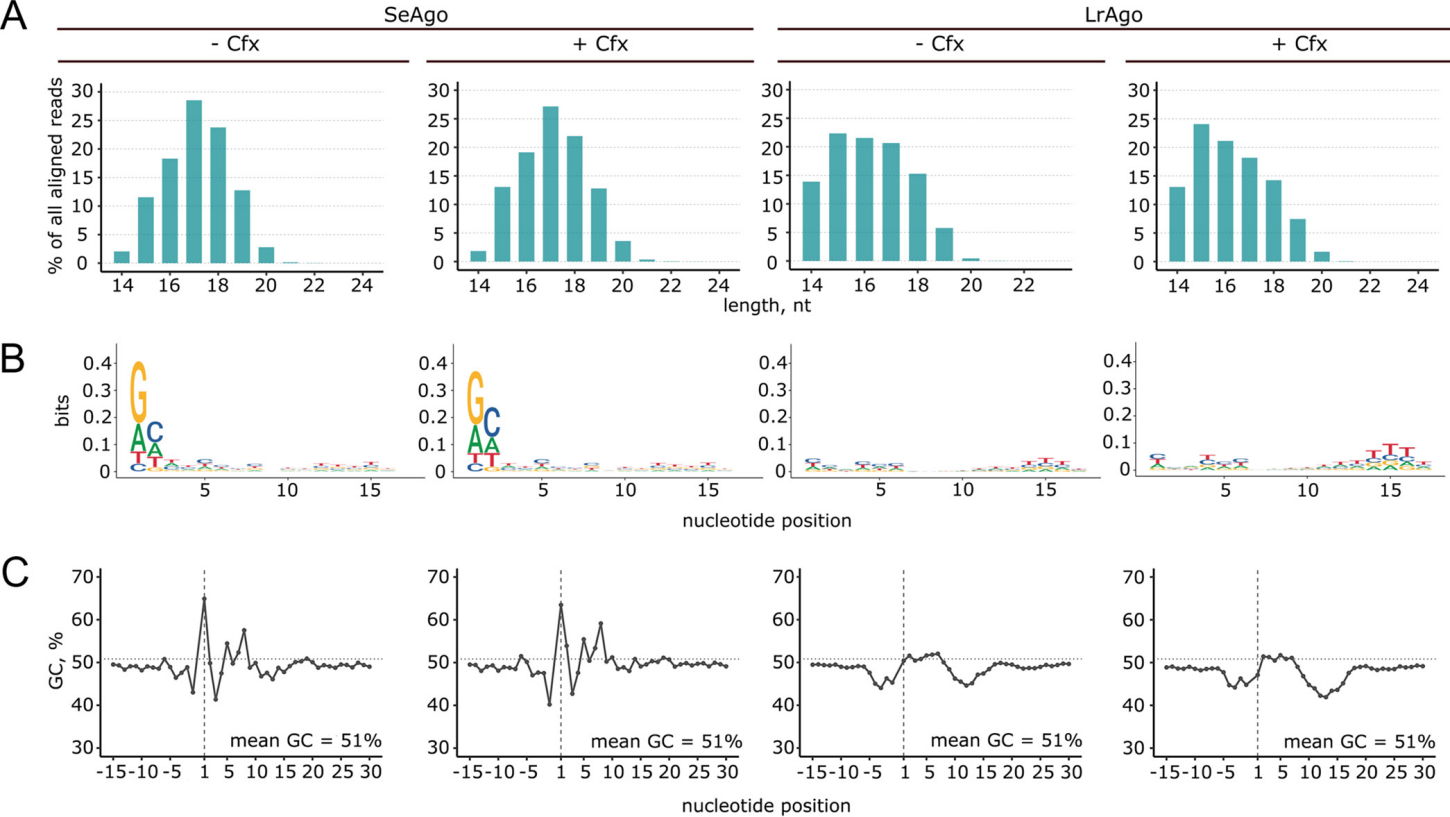
Characteristics of smDNAs associated with pAgos in E. coli in the absence or in the presence of Cfx. The smDNA samples were isolated at the exponential phase of growth (5.5 h) (see Fig. S4 in the supplemental material for the growth curves). (A) Distribution of smDNA lengths for each smDNA library. (B) Nucleotide logos for different smDNA positions starting from the 5′-end of guide DNA. (C) GC content of the smDNA sequences and of the surrounding genomic regions for each condition. The mean GC content of the E. coli genome is indicated.

Sequence analysis of smDNAs confirmed that the majority of smDNA associated with SeAgo and LrAgo had a length of 15 to 19 nt and 14 to 19 nt, respectively (Fig. 5A). Except for a slight preference for G at the first guide position in the case of SeAgo, no strong nucleotide biases were found along the guide length (Fig. 5B). The mean GC content of smDNAs corresponded to genomic DNA of E. coli (51%), and it was only slightly increased at the first guide position for SeAgo and slightly decreased upstream of the guide 5′-end and around 10 to 15 guide nucleotides for LrAgo (Fig. 5C). Overall, this analysis suggests that both SeAgo and LrAgo have no specific motif preferences and can likely interact with guide DNAs of any sequence.

Similar to several previously studied pAgos, both SeAgo and LrAgo were enriched with smDNAs derived from plasmid DNA (11- to 14-fold enrichment over chromosomal DNA for SeAgo and 3- to 9-fold preference for LrAgo after accounting for the relative replicon lengths and the plasmid copy number, 12 for pBAD). A similar bias for plasmid DNA was observed in the presence of Cfx (see Table S1 in the supplemental material). Nevertheless, the majority of smDNA guides (78 to 95% in various experiments) (Table S1) were derived from the E. coli chromosome, indicating that genomic DNA can be efficiently targeted by both pAgos.

**FIG 6:**
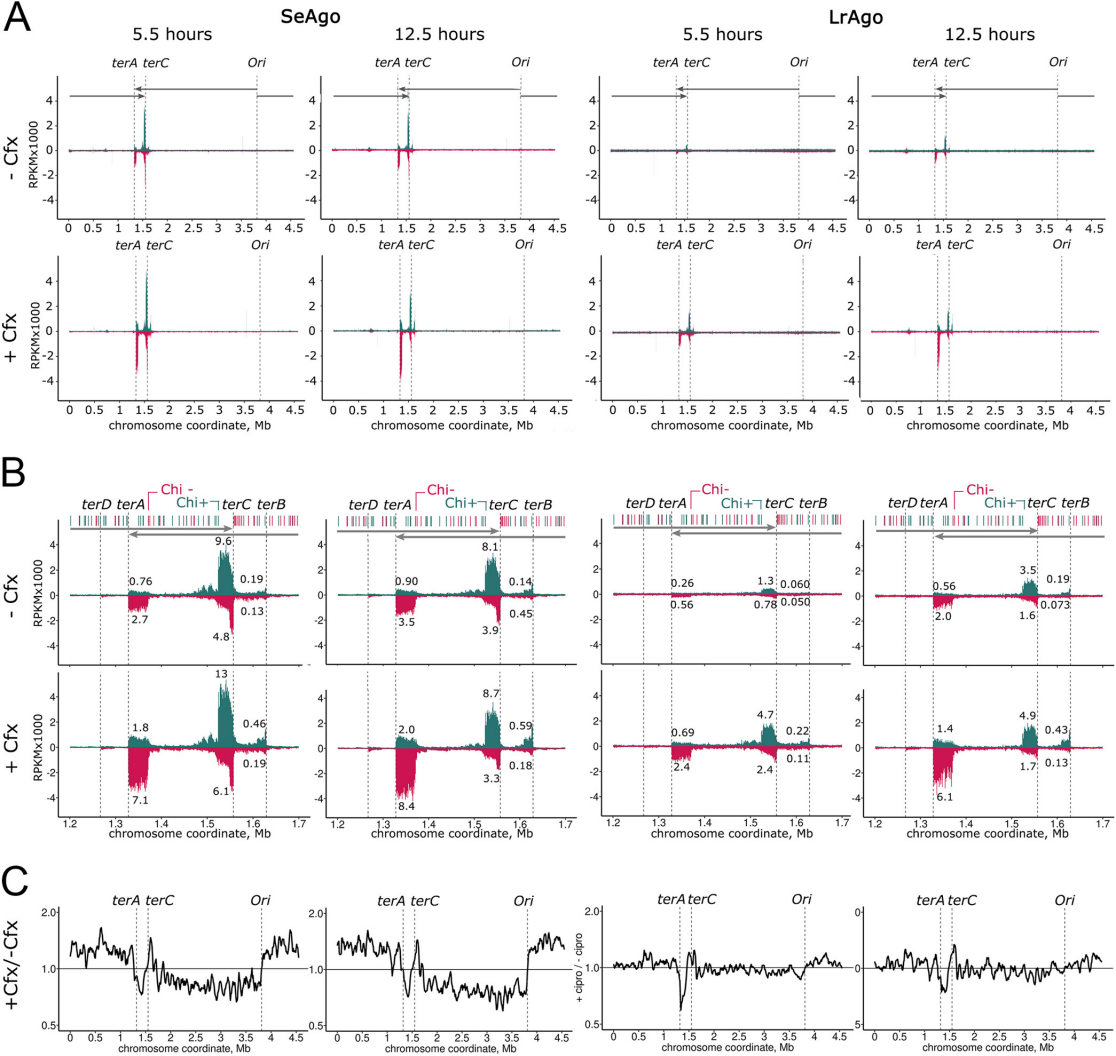
Whole-genome mapping of smDNAs associated with pAgos in E. coli. (A) Genomic distribution of smDNAs isolated from SeAgo and LrAgo in the absence or in the presence of Cfx at the logarithmic or stationary phases of growth (5.5 and 12.5 h) (see Fig. S4 in the supplemental material). The numbers of smDNAs along the genomic coordinate are shown in reads per kilobase per million aligned reads in the smDNA library (RPKM), individually for the plus (green) and minus (red) genomic strands. The *ori*, *ter* sites, and the direction of replication are indicated. (B) Targeting of the *ter* region by pAgos. SmDNA densities in each genomic strand (plus strand, green; minus strand, red) in the *ter* region in E. coli cultures grown in the absence (top) and in the presence (bottom) of Cfx are shown. Positions of the *terA*, *terC*, *terD*, and *terB* sites are shown with dashed lines and the directions of replichores are shown with arrows. Chi sites in the plus (green) and minus (red) strands are shown above the plots. The closest Chi sites oriented toward *terA* (“Chi−”), and *terC* (“Chi+”) are indicated. SmDNA numbers are shown in RPKM. The numbers of smDNAs from each DNA strand from each *ter* site, calculated for genomic regions between the *ter* site and the closest Chi site in the correct orientation, are shown as a percentage of the total number of smDNAs mapped to both strands of the whole genomic sequence in each smDNA library. (C) Effects of Sfx on the distribution of pAgo-associated smDNA between genomic DNA strands during replication. The ratio of pAgo-associated smDNAs corresponding to the plus and minus genomic strands was calculated independently for samples isolated from strains grown in the presence and in the absence of Cfx, and the obtained profiles were then divided by each other.

The distribution of smDNA guides along the chromosome was highly uneven, with two large peaks at the *terA* and *terC* sites of replication termination observed for both pAgos (Fig. 6A and B). In addition, two smaller peaks were present at the next pair of *ter* sites, *terB* and *terD* (Fig. 6B). For SeAgo, similar targeting of the *ter* sites was observed at different stages of cell growth. For LrAgo, targeting of the *ter* sites was less efficient at the logarithmic stage but was increased in the stationary culture (Fig. 6B).

The outer edges of the smDNA peaks precisely correspond to the *ter* motifs in chromosomal DNA bound by the Tus protein (Fig. 6B). The inner borders of the peaks coincide with the closest Chi-sites co-oriented with the direction of replication for each replichore (forward site in the plus strand for *terC* and reverse site in the minus strand for *terA*). Chi-sites (5′-GCTGGTGG-3′ in E. coli) serve as stop-signals for the RecBCD helicase-nuclease during processing of double-strand DNA breaks (DSBs), located downstream (in the 3′-direction) of the Chi sequence (36–38). This suggests that smDNAs in the *ter* region are produced with participation of RecBCD from double-strand DNA ends that are formed after stalling of the replication forks at Tus-bound *ter* sites.

In a further support for this notion, the ratio of smDNAs loaded from the plus and minus genomic strands in the *ter* region is asymmetric depending on the orientation of the *ter* sites. For both *terA* and *terC* (and *terB*), more smDNAs correspond to the DNA strand with the 3′-end oriented toward the *ter* site (the minus genomic strand for *terA* and the plus strand for *terC*; 2- to 3-fold more than for complementary 5′-terminated DNA strands). This corresponds to the asymmetry in smDNA processing by RecBCD previously reported for another pAgo protein, CbAgo (34).

Targeting of the *ter* region by both SeAgo and LrAgo was increased in the presence of Cfx (Fig. 6A and B, compare upper and lower rows). Specifically, the fraction of smDNAs corresponding to the peak at *terA* was increased ~2.6-fold and 3.9-fold for SeAgo and LrAgo, respectively, at the logarithmic stage of growth, and 2.4-fold and 2.9-fold at the stationary stage of growth (Fig. 6B). Furthermore, Cfx changed the relative sizes of the smDNA peaks at *terA* and *terC*. In the absence of Cfx, the peak at *terC* was larger than at *terA* for both pAgos at both stages of growth (Fig. 6B). This corresponds to the shorter length of the rightward replichore that terminates at *terC* and indicates that smDNAs at *ter* sites are likely produced during replication termination, which is more frequent at *terC* (34, 40). In contrast, the peaks at *terA* and *terC* became comparable in the presence of Cfx because smDNA loading was stimulated to a higher extent at *terA* than at *terC* (Fig. 6B). For both pAgos, this effect became especially prominent in the stationary cultures. This indicates that the relative frequencies of replication termination at *terA* and *terC* may be leveled in the presence of Cfx or that the efficiency of smDNA processing in these regions may become less dependent on replication in these conditions.

### Effects of ciprofloxacin-induced double-strand breaks on generation of pAgo-associated smDNAs.

To explore a possible connection between smDNA processing and replication on the whole-genome level, we compared the ratio of pAgo-bound smDNAs produced from the plus and minus genomic strands in the presence or in the absence of Cfx (Fig. 6C). In the case of SeAgo (but not LrAgo), Cfx increased generation of smDNAs from the leading DNA strand in both replichores, resulting in an increased ratio (>1) of smDNAs corresponding to the plus and minus strands for the rightward replichore and a decreased ratio (<1) for the leftward replichore (Fig. 6C). The only exception was the *ter* region, in which this ratio was changed in favor of the 3′-terminated strand at each *ter* site for both pAgos (<1 for *terA* and >1 for *terC*) as discussed in the previous section. Preferential generation of smDNAs from the leading DNA strand observed for SeAgo correlates with preferential co-orientation of Chi sites with the direction of replication and indicates that smDNAs may be produced during processing of double-strand breaks after gyrase inhibition and replisome stalling.

**FIG 7:**
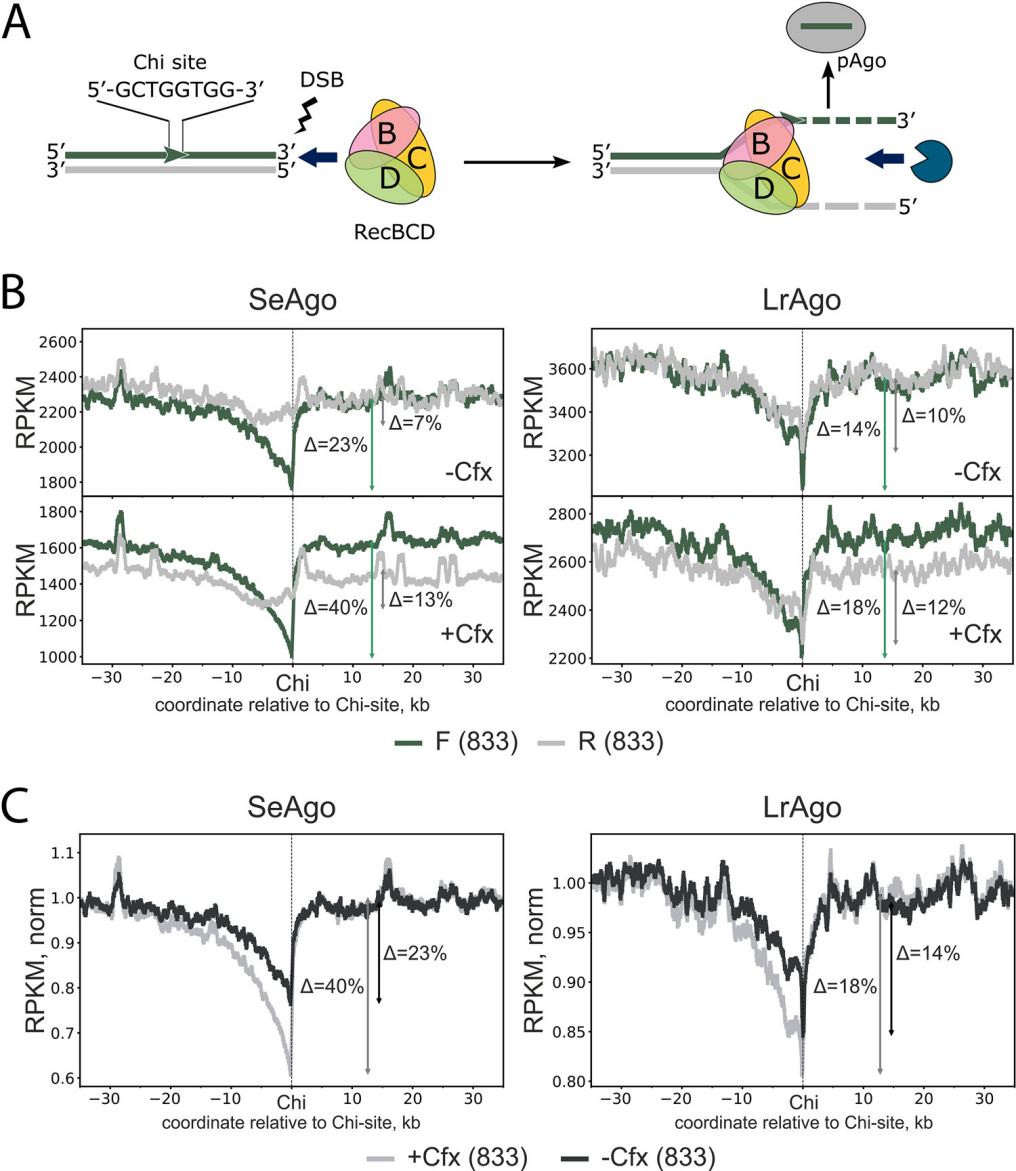
Asymmetry of smDNA distribution around Chi sites. (A) Scheme of DNA processing by RecBCD between a DSB and an upstream Chi site, illustrating the observed polarity of smDNA loading into pAgos. DNA unwinding by RecBCD is followed by asymmetric processing of the two DNA strands by RecBCD and other nucleases; RecBCD loads RecA onto the 3′-terminated strand at the Chi site, thus shielding this strand from further degradation. (B) Metaplots of the densities of smDNAs around Chi sites analyzed for smDNA libraries isolated from logarithmic cultures (5.5 h of growth) of E. coli strains grown in the absence and in the presence of Cfx (averages from two replicate experiments). SmDNA numbers were independently calculated for the DNA strands co-oriented (green, F) and oppositely oriented (gray, R) with the Chi sequence (5′-GCTGGTGG-3′) for all Chi sites in both genomic strands (833 sites in total) and smoothened with a 400-bp sliding window. (C) Comparison of metaplots of normalized densities of smDNAs around co-oriented Chi sites for smDNA libraries isolated from E. coli grown in the absence and in the presence of Cfx (the data correspond to green smDNA profiles in panel A). For normalization, averaged and smoothened RPKM values around Chi sites were divided by the background RPKM value calculated for regions remote from Chi sites (−50 to −35 kb and +35 to +50 kb from the Chi sequence). Arrows indicate relative differences between background and minimal densities of smDNAs at the Chi sites.

To further explore the role of double-strand DNA breaks (DSBs) and the RecBCD machinery in the biogenesis of smDNA guides bound by pAgos, we calculated the distribution of smDNAs around Chi sites throughout the whole chromosome, excluding the *ter* region (Fig. 7). This metaplot analysis revealed that distribution of pAgo-bound smDNA guides was asymmetric and dependent on the orientation of genomic DNA strands relative to the Chi sites. For the DNA strand co-oriented with Chi (forward orientation of Chi [F]), the amounts of smDNAs derived from the 3′-side of Chi were much higher than those from the 5′-side, with an abrupt drop immediately at the Chi sequence (Fig. 7A, B, green; see also Fig. S5A in the supplemental material). In particular, for SeAgo, this drop corresponded to 23% and 22% changes in the amounts of smDNAs at Chi sites relative to background levels in the exponential and stationary cultures, respectively. For the DNA strand that was oriented in the opposite direction relative to Chi (reverse orientation [R]), the changes in the amounts of smDNAs around Chi were much less pronounced (7% and 8% for SeAgo) (Fig. 7B, light gray; see also Fig. S5A). This indicates that recognition of Chi sites by RecBCD during processing of DSBs inhibits generation of smDNAs from the 3′-terminated DNA strand.

Inhibition of gyrase with Cfx stabilizes DSBs by covalently trapping cleaved DNA strands with the gyrase. This should increase RecBCD-dependent processing of gyrase-dependent DSBs and further stimulate biogenesis of smDNAs at Chi sites. Indeed, the asymmetry of smDNA loading around Chi sites was increased in the presence of Cfx for both pAgos at both exponential and stationary stages. In particular, the drop in the amounts of smDNAs at the properly oriented Chi sites was changed from 23% to 40% for SeAgo and from 14% to 18% for LrAgo in the logarithmic phase of growth (Fig. 7B and C) and from 22% to 40% for SeAgo and from 13% to 27% for LrAgo in the stationary phase (Fig. S5A and B). Thus, Cfx stimulates RecBCD-dependent processing of smDNAs, likely by increasing the number of DSBs formed in the chromosomal DNA due to inhibition of gyrase.

**FIG 8:**
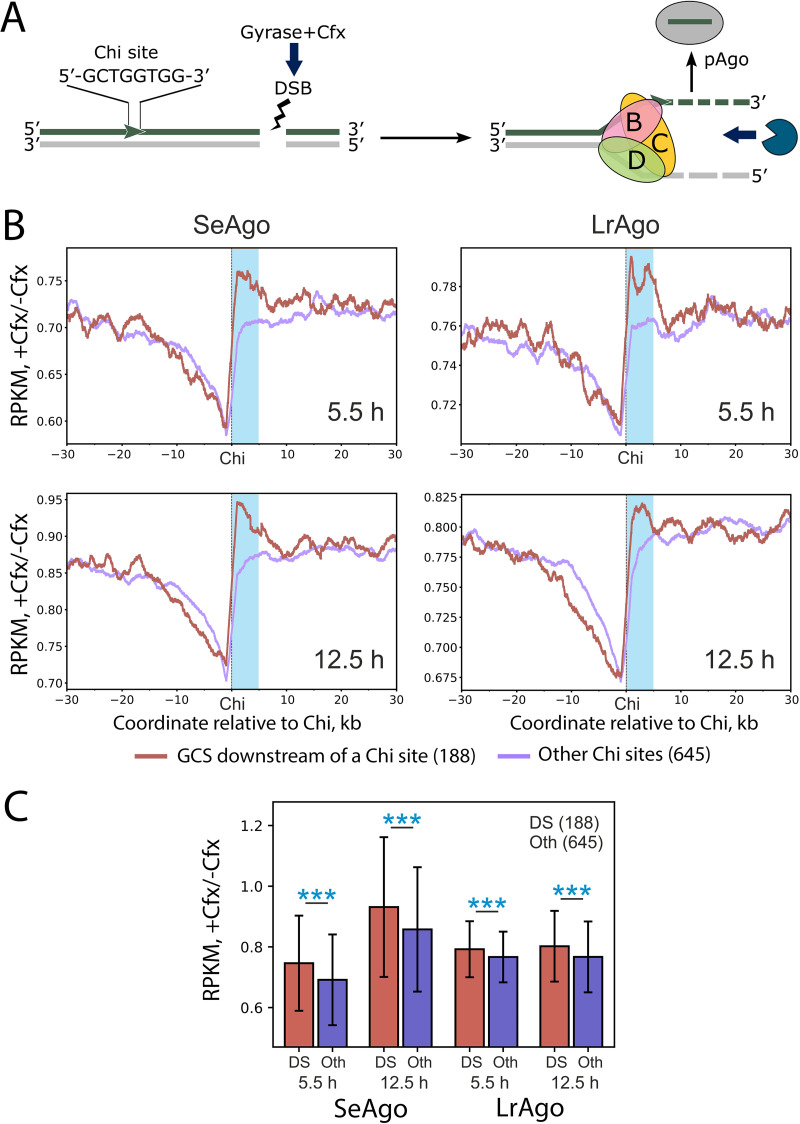
Effects of Cfx on the enrichment of pAgo-associated smDNAs around Chi sites with an adjacent GCS at the 3′-side. (A) Scheme of DNA processing between a GCS and the closest upstream Chi site, illustrating the observed polarity of smDNA loading into pAgos. (B) Metaplots of the relative densities of smDNAs around Chi sites adjacent to a downstream GCS from the 3′-side of the Chi sequence (carmine curves, 188 sites in total) and all other Chi sites, lacking adjacent downstream GCSs (lilas curves, 645 sites in total). A Chi site and a GCS were considered adjacent if there were no co-oriented Chi sites in between. The data were averaged for two replicate experiments for the exponential (5.5 h, top) and early stationary (12.5 h, bottom) phases of growth. SmDNA density was calculated for DNA strands co-oriented with the Chi sites for +Cfx and −Cfx conditions independently, and then +Cfx density was divided by Cfx density. The resultant relative density was smoothened with a 1-kb sliding window. Blue rectangles mark the region (from the Chi sequence to +5 kb) used to quantify the relative enrichments of smDNAs in panel B. (C) Quantification of relative enrichments of smDNAs at the 3′-sides of Chi sites for Chi sites adjacent to a downstream GCS (DS, carmine) and all other Chi sites (Oth, violet). The error bars represent mean values ± standard deviation (SD) for the same sets of Chi sites as in panel A. The enrichments were compared by a two-sided *t* test. *P* values of <0.0005 are indicated with three asterisks.

To directly test the effects from Cfx-induced DSBs on smDNA abundance, we analyzed the distribution of pAgo-associated smDNAs relative to Cfx-stabilized gyrase cleavage sites (GCSs), which were previously mapped in the E. coli genome (41). No enrichment of smDNAs was seen immediately at GCSs in Cfx-treated versus untreated cells (see Fig. S6 in the supplemental material). However, when we compared Chi sites with an adjacent GCS located downstream of the Chi sequence (Fig. 8A) and all other Chi sites with no adjacent downstream GCSs, a highly significant increase in smDNA amounts was observed for the first group of Chi sites (Fig. 8B). Specifically, the amounts of smDNAs were increased from the 3′-side of the Chi sequence, directly suggesting that gyrase-induced DNA cleavage stimulates smDNA processing between the GCS and the upstream Chi site. This increase was highly statistically significant for both pAgos and both stages of growth and was especially pronounced for SeAgo (Fig. 8C) (*P* values of 1.4e−5 and 2.9e−5 for 5.5 and 12.5 h, respectively). To confirm that the position of the GCS relative to the Chi sequence was important for smDNA processing, we also compared Chi sites with an adjacent downstream GCS (Chi sites in the active orientation) versus Chi sites with an adjacent upstream GCS (Chi sites in the inactive orientation). As expected, the enrichment of smDNAs at the 3′-side of Chi sites from the first group was significantly higher than in the case of Chi sites with upstream GCSs (see Fig. S7 in the supplemental material). Taken together, our data indicate that pAgos preferentially load smDNAs generated with participation of RecBCD at the 3′-sides of Chi sites during processing of downstream DSBs generated by Cfx.

### Distribution of pAgo-associated smDNAs relative to coding regions.

*In vitro* experiments with several pAgo proteins, including TtAgo, CbAgo, and LrAgo, demonstrated that their ability to process double-stranded DNA depends on DNA supercoiling (25, 27, 32). Our results indicate that gyrase inhibition, which induces DSBs and changes the supercoiling state of the chromosome, also changes the distribution of smDNAs. Another factor that could potentially affect smDNA processing by changing DNA supercoiling is transcription, which induces positive supercoils in front of the moving RNA polymerase and negative supercoils behind it (41–44). To explore the role of transcription in the targeting of chromosomal DNA by pAgos, we first compared the abundances of pAgo-associated smDNAs derived from each genomic strand for genes codirected or oppositely directed relative to replication. No differences between these groups of genes were found for either SeAgo or LrAgo (see Fig. S8A in the supplemental material). Divergent and convergent gene orientation is associated with negative and positive DNA supercoiling in intergenic regions, respectively, which could affect smDNA production (42). We analyzed smDNA abundance in intergenic DNA regions for divergent, convergent, and co-oriented gene pairs. No significant differences in the amounts of smDNAs were detected for the different types of gene pairs for both pAgos (Fig. S8B). Together, our data suggest that transcription-induced supercoiling is unlikely to have major effects on smDNA biogenesis.

### Analysis of the effects of ciprofloxacin and SeAgo on cell division in *S. elongatus*.

The experiments presented above demonstrated that pAgo proteins from mesophilic cyanobacteria can suppress defects in DNA replication in E. coli caused by gyrase inhibition, with stronger effects observed for SeAgo. To test whether SeAgo may have similar functions in its native species, we compared *S. elongatus* strains with the natural level of expression of SeAgo (wild-type strain), without SeAgo (ΔSeAgo), and with an increased level of SeAgo, expressed from a strong constitutive promoter (↑SeAgo). Titration of Cfx demonstrated that growth of all three strains in liquid culture was fully inhibited at ≥15 ng/mL of the antibiotic, indicating that *S. elongatus* is slightly less sensitive to Cfx than E. coli (see Fig. S9A in the supplemental material). We then compared cell growth at sublethal Cfx concentrations. Wild-type and ΔSeAgo strains had identical growth kinetics in the absence and in the presence of 10 ng/mL of Cfx (Fig. S9A). In contrast, the growth of the strain with overexpression of SeAgo was strongly inhibited in the presence of the same Cfx concentration. Microscopy analysis revealed no changes in the cell number or morphology in either wild-type or ΔSeAgo strains in these conditions (Fig. S9B). Cell morphology also remained unchanged in the case of the overexpressor strain, despite the lower number of cells observed in the presence of Cfx (Fig. S9B). Thus, SeAgo does not increase the resistance of the wild-type strain of *S. elongatus* to Cfx in comparison with the deletion strain, while its overexpression is toxic in the presence of the antibiotic. Furthermore, Cfx inhibits *S. elongatus* growth without formation of filamentous cells, suggesting that its mechanism of action in cyanobacterial cells may be different from E. coli.

To get further insight into possible functions of SeAgo in *S. elongatus*, we compared sensitivity of *S. elongatus* strains with various levels of expression of SeAgo to other genotoxic agents, 4-nitroquinoline 1-oxide (4-NQO) and nitrofurazone (NFZ). Both compounds produce DNA guanosine adducts that are mainly removed by nucleotide excision repair but may also induce secondary DNA damage and DSBs during replication (45–47). It was found that strains with wild-type SeAgo, deletion of SeAgo, overexpression of wild-type SeAgo, and overexpression of a catalytically dead variant of SeAgo all had similar sensitivities to these treatments (see Fig. S10 in the supplemental material). Therefore, the primary function of SeAgo in *S. elongatus* may not be related to DNA replication and cell division (see Discussion).

## DISCUSSION

In contrast to eAgos that recognize RNA targets, the majority of studied pAgos preferentially target DNA *in vitro* and *in vivo*, suggesting that their mechanism of action is different from eAgos. However, similarly to eAgos, studied pAgos can target foreign genetic elements (25, 29, 31–34), demonstrating that the defensive function of Ago proteins is conserved between prokaryotes and eukaryotes (48). At the same time, pAgos might play other roles, including the regulation of gene expression, participation in cellular suicide systems, and DNA repair (21, 29, 49–52). In particular, TtAgo from T. thermophilus was recently shown to participate in separation of chromosomal DNA during replication (35). This activity of TtAgo became crucial for cell division when the DNA gyrase, the sole type II topoisomerase in T. thermophilus, was inhibited by Cfx. TtAgo associated with small guide DNAs corresponding to the termination region of replication and coprecipitated with several proteins involved in DNA processing, including gyrase and factors involved in DNA recombination (AddAB, a RecBCD homolog in T. thermophilus). It was therefore proposed that TtAgo might help to decatenate daughter chromosomes by direct DNA cleavage and/or by recruiting accessory factors to the termination region (35). However, suppressive effects of TtAgo were not observed for another DNA gyrase inhibitor, novobiocin, which prevents DNA cleavage by gyrase without producing DSBs in chromosomal DNA (35). This suggests that, alternatively, TtAgo may be involved in the repair of DSBs at GCSs induced by Cfx.

Here, we have demonstrated that two pAgo proteins from mesophilic cyanobacteria, SeAgo and LrAgo, facilitate cell division and prevent cell filamentation in the presence of Cfx in E. coli. The primary target of Cfx in E. coli is gyrase (53–56), and both pAgos also suppress a milder phenotype caused by dCas9 knockdown of gyrase expression in E. coli. Inhibition of gyrase by Cfx strongly affects replication by changing DNA supercoiling and integrity and by introducing direct roadblocks to the moving replisomes (56–59). Additionally, Cfx can target topoisomerase IV in E. coli, which is also involved in chromosome decatenation (55). Thus, formation of cell filaments with unsegregated DNA in the presence of Cfx can be explained by failure to separate daughter chromosomes due to their incomplete replication and decatenation and by the induction of the SOS response after DNA damage, preventing cell division (59–64).

**FIG 9:**
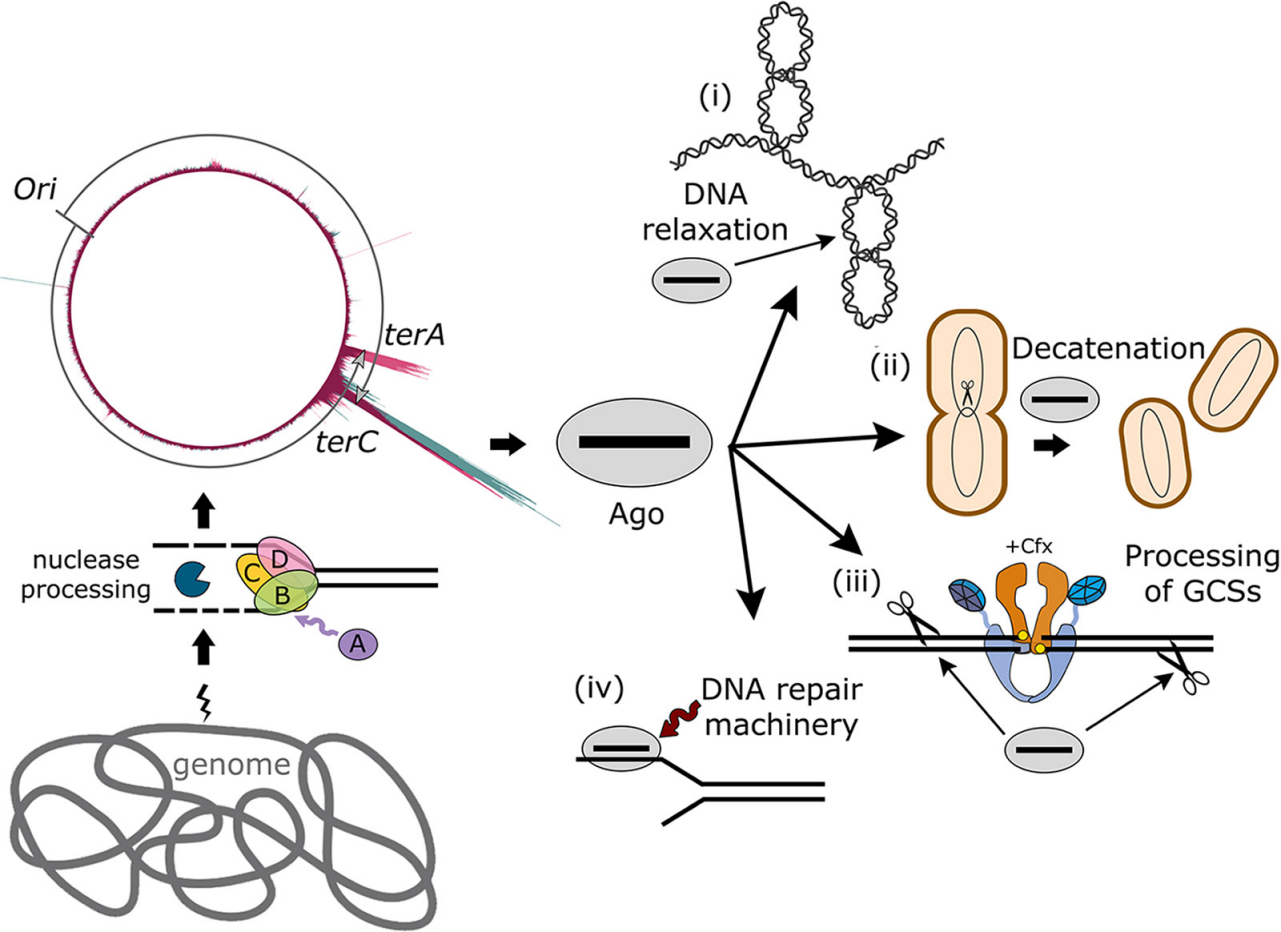
Possible activities of pAgos explaining their observed suppressor effects on bacterial cell growth. pAgos capture guide smDNAs generated during chromosomal DNA replication and repair with the participation of RecBCD. Guide-loaded pAgo may then (i) relax supercoils in the replicating chromosome through its DNA-nicking activity, (ii) help to decatenate chromosomes under conditions of gyrase inhibition by attacking both DNA strands with guide DNAs corresponding to the *ter* region or by processing of nicked DNA strands, (iii) participate in the processing of DSBs generated after gyrase inhibition by Cfx and possibly facilitate removal of covalently bound gyrase, or (iv) recruit additional factors involved in DNA processing and repair (as proposed for TtAgo [35]).

Available data suggest several possible mechanisms explaining the observed effects of pAgos on DNA replication and cell division. The patterns of smDNA loading into both SeAgo and LrAgo suggest that genomic smDNAs are primarily generated during nuclease-dependent processing of DSBs and replication intermediates (Fig. 9). When loaded with smDNAs, SeAgo, and LrAgo may help the cells to complete DNA replication by direct targeting of chromosomal DNA through their nuclease activity. By doing this, they may help to (i) relax excessive supercoils accumulated in the absence of gyrase by their DNA nicking activity, and (ii) decatenate sister chromosomes by introducing DSBs in the *ter* region. Cleavage of both DNA strands may be performed by pAgos loaded with complementary smDNAs produced from the *ter* sites or by their binding to pre-existing nicks and cutting the second DNA strand as previously proposed for TtAgo (35). Alternatively, (iii) pAgos may assist the cellular DNA repair machinery in the removal of covalent gyrase-DNA intermediates stabilized by Cfx or in the processing of the resulting DSBs, thus accelerating the repair process. This mechanism is supported by the observed enrichment of smDNAs from the 3′-sides of Chi sites adjacent to GCSs in Cfx-treated cells. Finally, (iv) TtAgo was proposed to recruit DSB repair factors to the *ter* region in T. thermophilus, possibly facilitating DNA repair even without pAgo-dependent DNA cleavage (35). However, SeAgo and LrAgo are not expected to specifically interact with host-specific factors in heterologous E. coli and are more likely to directly participate in chromosomal DNA processing. Indeed, the catalytic activity of pAgos contributes to smDNA biogenesis since a catalytically dead mutant of SeAgo does not bind smDNAs in E. coli (28), and much lower amounts of smDNAs are associated with a catalytically dead mutant of TtAgo in T. thermophilus. The catalytic activity of pAgos is also important for their protective function since mutant TtAgo provides only low levels of protection against Cfx in T. thermophilus (35).

Both SeAgo and LrAgo preferentially target the replication termination region of the E. coli chromosome and are loaded with smDNAs corresponding to the *ter* sites. Similar enrichment of pAgo-associated smDNAs at the *ter* sites was previously observed for TtAgo in T. thermophilus (35) and for CbAgo expressed in E. coli (34). SmDNAs loaded into pAgos in the *ter* region are preferentially generated from 3′-ends of DNA strands oriented toward *ter* sites and are confined to the areas between *ter* sites and the closest Chi sites. SmDNA loading into pAgos also depends on Chi sites on the genomic scale and is not restricted to the *ter* region. This pattern indicates that smDNAs are produced during asymmetric processing of the two DNA strands at replicated DNA ends or DSBs by RecBCD and/or other cellular nucleases that cooperate with RecBCD during DNA unwinding (34, 36–38).

The proposed role of pAgos in DNA decatenation requires introduction and repair of DSBs in chromosomal DNA. Indeed, the anti-inhibitory function of pAgos depends on the homologous recombination machinery and is not observed in E. coli strains lacking the key components of double-strand break repair, RecBCD or RecA. As previously shown for CbAgo, RecBCD is not required for biogenesis of smDNAs and their loading into pAgos; however, inactivation of RecBCD changes the pattern of chromosomal smDNA distribution and removes smDNA peaks around *ter* sites (34). Thus, it remains to be established whether the absence of protective activity of SeAgo and LrAgo in *rec*-minus strains may result from changes in smDNA processing or from defects in DSB repair caused by inactivation of RecBCD.

The asymmetry of smDNA processing in the *ter* region and around Chi sites on the chromosomal scale are both increased in the presence of Cfx, likely as a result of increased levels of gyrase-mediated DNA fragmentation induced by the antibiotic. Indeed, this increase is primarily explained by stimulation of DSB formation downstream of Chi sites at GCSs by Cfx. This may result in increased targeting of the *ter* region and GCSs by guide-loaded pAgos and help to overcome problems with chromosomal DNA processing during replication. In support of this, the stronger effects of SeAgo on cell growth and morphology in the presence of Cfx correlate with its higher loading with smDNAs from the *ter* sites and a stronger asymmetry of smDNA processing around Chi sites in comparison with LrAgo. At the same time, the ability of SeAgo and LrAgo to suppress the effects of mild gyrase knockdown, which is not associated with Cfx-induced DNA cleavage, suggests that they may have pleiotropic effects on DNA processing. Further research is needed to decipher the interplay between pAgos and the recombination machinery in various species and to elucidate the possible roles of pAgos in DNA replication, decatenation, or repair (Fig. 9).

Strikingly, while SeAgo protects E. coli cells from the toxic effects of Cfx, it does not protect its host strain of *S. elongatus*. Moreover, overexpression of SeAgo makes *S. elongatus* more susceptible to this antibiotic. This may be explained by differences in chromosomal DNA targeting by SeAgo and/or by differences in the mechanism of inhibition of cell division by Cfx in cyanobacteria and in E. coli. Indeed, a different pattern of SeAgo-associated smDNAs was observed for SeAgo in its native host *S. elongatus*, with no major peaks in the predicted termination region (28). While *S. elongatus* encodes several topoisomerases including Topo I, Topo II, and gyrase, it lacks recognizable *ter* sites in the chromosome and its mechanisms of replication termination remain poorly understood (65). Similarly to T. thermophilus, *S. elongatus* encodes the RecBCD homolog AddAB, but its potential Chi sites have not been identified in the genome of *S. elongatus* or other cyanobacteria (66, 67). Thus, the specificity of chromosomal DNA targeting by different pAgos may be different in various species depending on the mechanisms of DSB processing and replication termination. Furthermore, cyanobacteria do not have classical SOS response characterized in E. coli, and *S. elongatus* apparently lacks its master regulator, the LexA repressor (68, 69). Accordingly, cell filamentation was not observed in cyanobacteria (*S. elongatus* or *Synechocystis*) treated with Cfx or other DNA-damaging agents (70–72). At the same time, changes in the supercoiling state of the chromosome caused by gyrase inhibition in cyanobacteria were shown to induce major changes in the expression of genes involved in adaptation and to impair stress response (70, 73). Thus, by acting as a DNA endonuclease, SeAgo may additionally increase DNA damage rather than aid DNA processing in *S. elongatus* cells treated with Cfx.

The natural functions of SeAgo in cyanobacterial cells remain to be investigated. We have previously shown that its deletion or overexpression do not affect the kinetics of cell growth under laboratory conditions (28). Furthermore, SeAgo does not change the sensitivity of *S. elongatus* to other DNA-damaging agents tested here. At the same time, loss of function of SeAgo was shown to increase the efficiency of plasmid DNA transfer in *S. elongatus* (74, 75). SeAgo may therefore modulate natural transformation in its host bacterium by targeting and processing of foreign DNA. At the same time, CbAgo was recently shown to stimulate homologous recombination in E. coli by inducing DSBs in chromosomal DNA, suggesting that pAgos may promote insertion of homologous sequences into the chromosome (76, 77). Further research is needed to understand possible roles of SeAgo in its host species, including natural competence and homologous recombination. Importantly, our findings suggest that the functions of pAgo proteins may change upon their transfer between prokaryotic species (1, 2, 4) and may switch between cell protection, regulation of horizontal gene transfer, and processing and repair of chromosomal DNA.

## MATERIALS AND METHODS

### Plasmids and strains.

All E. coli strains were isogenic to E. coli BL21(DE3). *recA*-minus and *recBD*-minus strains were obtained previously (34). E. coli cells were routinely cultivated in LB Miller broth (2% tryptone, 0.5% yeast extract, 1% NaCl, pH 7.0) with the addition of ampicillin (Amp) (100 μg/mL) if needed. The gene of SeAgo (Protein database accession number WP_011378069.1) was codon-optimized using IDT Codon Optimization Tool for expression in E. coli, synthesized by the IDT core facility, and cloned into pBAD-HisB in frame with an N-terminal His_6_ tag. The pBAD-HisB plasmid encoding LrAgo was obtained previously (27). E. coli strains were transformed with pBAD plasmids encoding each of the two pAgos or a control plasmid without pAgo, grown overnight in LB with Amp, diluted twice with 50% glycerol, aliquoted, frozen in liquid nitrogen, and stored at −80°C.

### Ciprofloxacin treatment and analysis of growth kinetics in E. coli.

To determine the range of inhibitory concentrations of Cfx and analyze the effects of pAgos on the growth kinetics, overnight bacterial cultures of E. coli BL21(DE3) carrying the control pBAD vector or pBAD-encoding pAgo genes were obtained from the frozen aliquots and inoculated into 200 μL of fresh LB medium supplemented with a range of Cfx concentrations (from 62.5 ng/mL to 0.2 ng/mL obtained by 2-fold serial dilutions; 0.5 ng/mL for comparison of wild-type and mutant strains) or without Cfx, without or with 0.01% l-arabinose (Ara), and with Amp in 96-well plates (TPP; flat bottom). The plates were incubated at 300 rpm at 30°C in a SPECTROstar Nanomicroplate reader, and cell density was monitored by measuring OD_600_ every 10 min. Three independent biological replicates were performed in each case. Additionally, after 4.5 h, cells were harvested for Western blot analysis, microscopy, and determination of CFU numbers. To calculate CFU, serial dilutions of the culture were plated on selective agar medium containing Amp.

### Western blotting.

The levels of SeAgo and LrAgo expression were determined by Western blotting. E. coli cells expressing pAgos were harvested by centrifugation, the pellet was mixed with 1× Laemmli sample buffer (120 mM Tris-HCl, 4% SDS, 4% β-mercaptoethanol, 10% glycerol, pH 6.8) and heated at 95°C for 5 min, and the samples were resolved by electrophoresis in a 4 to 20% Tris-glycine gel (Bio-Rad). Proteins were transferred onto a nitrocellulose membrane in Towbin transfer buffer (25 mM Tris, 192 mM glycine, 20% methanol) using semidry procedure at 25 V, 1 A for 30 min (Bio-Rad; Trans-Blot Turbo). The transfer membrane was washed in phosphate-buffered saline (PBS) (10 mM phosphate buffer, 137 mM NaCl, 2.7 mM KCl) for 5 min. The membrane was blocked with blocking buffer (PBS, Tween 20 0.1% [vol/vol], nonfat milk 5% [wt/vol]) for 30 min at room temperature, and then incubated with anti-His_6_ monoclonal antibodies (1:1,000; Sigma) for 1 h at room temperature. The membrane was washed four times with PBST buffer (PBS, Tween 20 0.1% [vol/vol]) and incubated with horseradish peroxidase (HRP)-conjugated anti-mouse secondary antibodies (1:10,000, Sigma) for 1 h at room temperature and washed again as described above. Antigen-antibody complexes were detected with Immobilon ECL Ultra Western HRP substrate (Millipore) on a ChemiDoc XRS+ imager (Bio-Rad).

### Preparative growth of E. coli and purification and sequencing of pAgo-associated smDNAs.

Overnight bacterial cultures were obtained from frozen aliquoted cultures and inoculated into 500 mL of fresh LB medium supplemented with 0 or 0.3 ng/mL Cfx, 0.01% Ara, and Amp in 2-L flasks. The flasks were incubated at 190 rpm at 30°C in an orbital shaker, and cell density was monitored by measuring OD_600_ every 30 min. Two independent biological replicates were performed. The cells were harvested by centrifugation at 7,000 × *g*, 4°C for 15 min after 5.5 and 12.5 h of growth for protein pulldown. The cells were disrupted with a high-pressure homogenizer (EmulsiFlex-C5; Avestin) at 18,000 lb/in^2^. pAgos were pulled down using Co^2+^-Talon metal affinity resin (TaKaRa) as described previously (29). Eluted proteins were treated with Proteinase K for 30 min at 37°C, and small nucleic acids were extracted with phenol-chloroform, ethanol precipitated, dissolved in water, and analyzed by PAGE as described (29).

Libraries for high-throughput sequencing of smDNAs were prepared according to the previously published splinted ligation protocol (29). Briefly, nucleic acids extracted from pAgos were treated with RNase A (Thermo Fisher), purified by PAGE, and small DNAs (14 to 20 nt) were eluted from the gel in 0.4 M NaCl overnight at 21°C, ethanol precipitated, dissolved in water, phosphorylated with polynucleotide kinase (New England Biolabs), and ligated with adaptor oligonucleotides using bridge oligonucleotides as described in reference 29. The ligated DNA fragments were purified by denaturing PAGE, amplified, and indexed by the standard protocol for small RNA sequencing (New England Biolabs). Small DNA libraries were sequenced using the HiSeq 2500 platform (Illumina) in the rapid run mode (50-nucleotide single-end reads). The list of all sequenced smDNA libraries is presented in Table S1.

### Analysis of chromosomal distribution of smDNAs.

Analysis of smDNA sequences was performed as described previously (34). After trimming the adaptors, reads shorter than 14 nt were removed with CutAdapt (v. 2.8). Bowtie (v. 1.2.3) was used to align the reads to the reference genomic DNA (RefSeq accession number NC_012971.2) with no mismatches allowed. Nucleotide logos were calculated using reads longer than 16 nt, and reads longer than 17 nt were truncated to 17 nt from the 3′-end. Genome coverage was calculated using BEDTools (v. 2.27.1) and custom Python scripts. Whole-genome coverage of each DNA strand was calculated in 1,000-nt windows and normalized by the total number of mapped reads in the library, expressed as RPKM (reads per kilobase per million aligned reads). To calculate the percent of reads mapped to the regions around *ter* sites, the number of smDNAs for each DNA strand from each region (from 1,328,000 to 1,350,000 for *terA*, from 1,524,000 to 1,557,000 for *terC*, and from 1,626,000 to 1,629,000 for *terB*) was divided by the total number of reads mapped to both strand of the genome. To determine the asymmetry of smDNA distribution between the two genomic strands depending on Cfx treatment, the ratio between the coverage of the plus and minus strands from the Cfx-treated cell culture was divided by the same ratio obtained for the control library and plotted as a rolling mean (50-kb window, 10-kb step).

To calculate smDNA distribution around Chi sites, GCSs, in genes, and intergenic intervals, the region of replication termination (1.2 to 1.7 Mb chromosomal coordinates) and several other highly enriched regions, including rRNA operons, were removed from the analysis (see the full list of masked regions in Table S2 in the supplemental material). Gene borders were extracted from the RefSeq annotation file. Intergenic intervals with lengths of <500 nt were classified into four groups depending on the relative orientation of the surrounding gene, and 100 nucleotides were additionally included from each side to each interval. SmDNA coverage of genes and intergenic intervals was calculated in RPKM. GCSs induced by Cfx were mapped in the reference genome essentially as described previously using Topo-Seq data obtained for E. coli with a 0.01 *P* value threshold for the Audic-Claverie test (41). In total, 1,931 GCSs were identified in the reference genome, 1,833 of which were considered for further analysis and 98 of which were mapped in excluded regions. To prepare metaplots with GCSs as anchors, smDNA densities were extracted for both DNA strands around GCSs (±15-kb region) for +Cfx and −Cfx conditions. SmDNA densities were averaged position-wise for the +Cfx and −Cfx data sets independently. The averaged +Cfx density was divided by the averaged −Cfx density, and the resultant relative density was smoothed with a 2-kb sliding window. Plots were generated in R (v. 3.6.3) using ggplot2 (v. 3.3.3) and ggseqlogo (v. 0.1) (78) libraries and in python (v. 3.7) using matplotlib (v. 3.5.1).

### Strains of *S. elongatus*, growth conditions, and ciprofloxacin titration.

Wild-type strain of *S. elongatus* PCC 7942 was obtained from Invitrogen. Strains with deletion and overexpression of the *ago* gene were obtained previously using standard protocols (28). Cyanobacterial strains were maintained in liquid BG11 with shaking or on solid BG11 plates (79) under constant light conditions (~250 μE m^−2^ s^−1^) at 30°C with 10 μg/mL spectinomycin if needed.

Dense cultures of *S. elongatus* wild-type and mutant strains were inoculated into 800 μL of fresh BG11 medium supplemented with 0 to 35 ng/mL Cfx and spectinomycin (in the case of the mutant strains) in 48-well plates (Eppendorf). The plates were incubated at 300 rpm at 30°C under constant light conditions, and cell density was monitored by measuring OD_750_ every 12 h. One biological replicate was performed for titration and three independent biological replicates were performed for the growth experiment with 0 and 10 ng/mL Cfx. Cells were harvested for microscopy 48 h after inoculation.

To analyze the effects of 4-NQO and NFZ on cell growth, serial dilutions of the wild-type strain of *S. elongatus* and strains with deletion of SeAgo and with overexpression of wild-type or catalytically dead SeAgo were plated on LB agar containing increasing concentrations of 4-NQO and NFZ (from 1 to 10 μM) and grown for 12 days at 30°C under constant light conditions.

### Cell microscopy.

E. coli cells were visualized using acridine orange staining. Sterilized slides were fixed with 95% ethanol for 2 min, excess ethanol was drained, and the slides were allowed to air dry. Bacterial culture was placed onto a slide, dried, and briefly fixed in the flame of a burner. The slide was flooded with acridine orange stain for 2 min, rinsed thoroughly with tap water, and allowed to air dry. Cell pictures were taken on a ZEISS LSM 900 confocal laser scanning microscope (Carl Zeiss) with a 63× oil immersion objective and with 100 μm confocal pinhole aperture. Cell pictures of *S. elongatus* were taken similarly but using chlorophyll autofluorescence, which was excited at 488 nm and recorded using a 650-nm long-pass filter. The obtained pictures were processed using the ZEN Microscopy software (Carl Zeiss).

### Gyrase knockdown with dCas9.

To knockdown the *gyrA* gene, a plasmid carrying the p15A origin, chloramphenicol resistance and dCas9 genes, and sgRNA was obtained by Gibson assembly. The 20-nt guide sequence in sgRNA corresponding to the *gyrA* gene (5′-AGCTCTTCCTCAATGTTGAC-3′) was chosen based on the algorithm developed in reference 80. E. coli Bl21(DE3) strains containing pBAD plasmids encoding or lacking pAgos were transformed with the dCas9 plasmid and grown under standard conditions with the addition of 0.01% Ara, Amp, and chloramphenicol. The drop of the *gyrA* expression level was measured by quantitative reverse transcription-PCR using primers corresponding to the *gyrA* gene (qPCR_gyrA_for 5′-TTATGACACGATCGTCCGTATG and qPCR_gyrA_rev 5′-TTCCGTGCCGTCATAGTTATC) and the *rpoB* gene for normalization (rpoB_Fw1 5′-ATGGTTTACTCCTATACCGAGAAAAAAC and rpoB_Rv1 5′-TATTGCAGCTCGGAATTACCG).

### Data availability.

The smDNA sequencing data sets generated in this study are available from the Sequence Read Archive (SRA) database under BioProject number PRJNA878808. The code used for data analysis is available at the GitHub repository at https://github.com/AlekseiAgapov/SeAgo_LrAgo. All primary data are available from the corresponding author upon request.
