# Supplementary material for: Bacterial Argonaute Proteins Aid Cell Division in the Presence of Topoisomerase Inhibitors in Escherichia coli
Source: Microbiol Spectr. 2023 Apr 27;11(3):e04146-22. doi: 10.1128/spectrum.04146-22 (PMC10269773; doi:10.1128/spectrum.04146-22)
Supplement: Supplemental file 1 — Supplemental material. Download spectrum.04146-22-s0001.pdf, PDF file, 6.2 MB [file spectrum.04146-22-s0001.pdf]

Bacterial Argonaute proteins aid cell division in the presence of topoisomerase inhibitors in *Escherichia coli*

Anna Olina, Aleksei Agapov, Denis Yudin, Dmitry Sutormin, Alina Galivondzhyan, Anton Kuzmenko, Konstantin Severinov, Alexei A. Aravin, Andrey Kulbachinskiy

SUPPLEMENTAL MATERIAL

Includes Figures S1-S10, Tables S1, S2

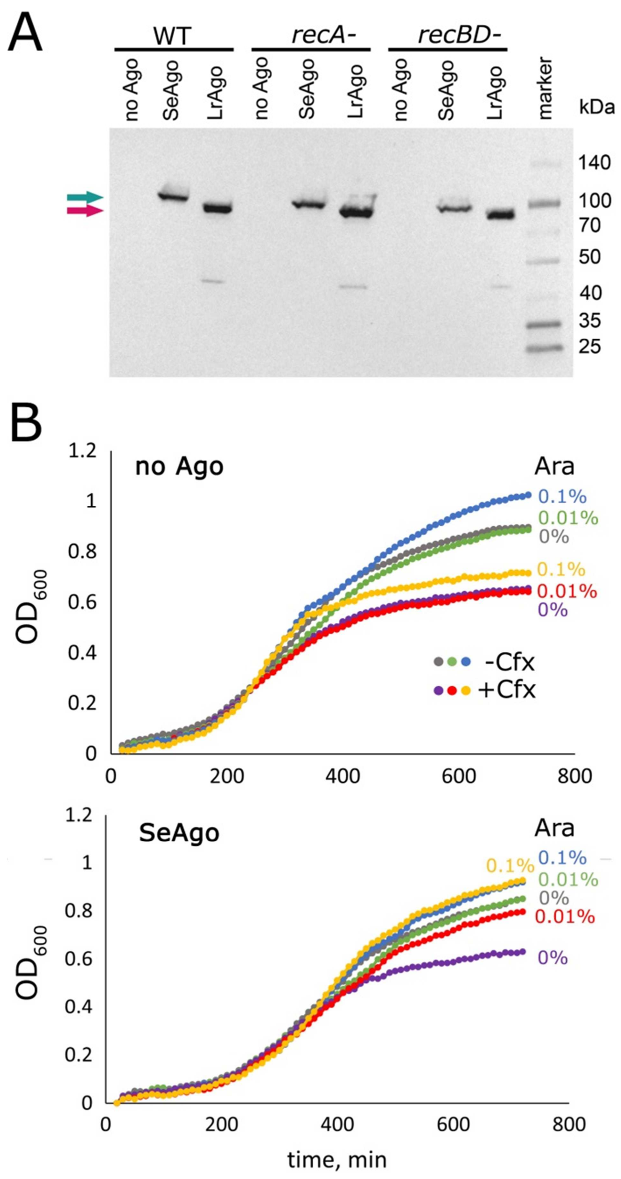

Fig. S1. Expression of SeAgo and LrAgo in *E. coli*. (A) Analysis of pAgo expression in *E. coli* by Western blotting. Protein samples were obtained from the wild-type or *rec*-minus *E. coli* strains at 4.5 hours of growth. The proteins were separated by 4-20% gradient denaturing SDS-PAGE, transferred to a nitrocellulose membrane and visualized by Western-blotting with His-tag specific antibodies (see Materials and Methods for details). The turquoise and pink arrows show positions of SeAgo and LrAgo, respectively. (B) Growth of *E. coli* strains in the absence or presence of Cfx depending on expression of SeAgo (uniduced, 0% Ara; induced with 0.01% or 0.1% of Ara). *E. coli* cells were transformed with empty pBAD or pBAD encoding SeAgo and were grown at 30 °C in a plate reader without Cfx or with Cfx (0.5 ng/mL) and indicated concentrations of arabinose. Averages from 3 technical replicates are shown.

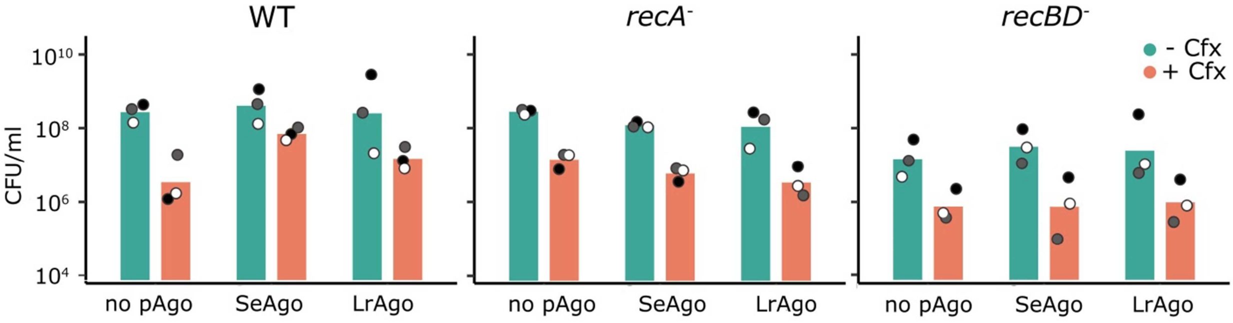

Fig. S2. Analysis of the CFU numbers for *E. coli* strains lacking or containing pAgos and grown in the absence and in the presence of Cfx. The samples were taken from *E. coli* cultures grown for 4.5 hours in the absence or in the presence of Cfx (Fig. 3A), and CFU numbers were determined by plating their serial dilutions on LB agar plates without Cfx. Means from three biological replicates are shown, individual data points from each measurement are indicated (colored pairwise for each independent experiment).

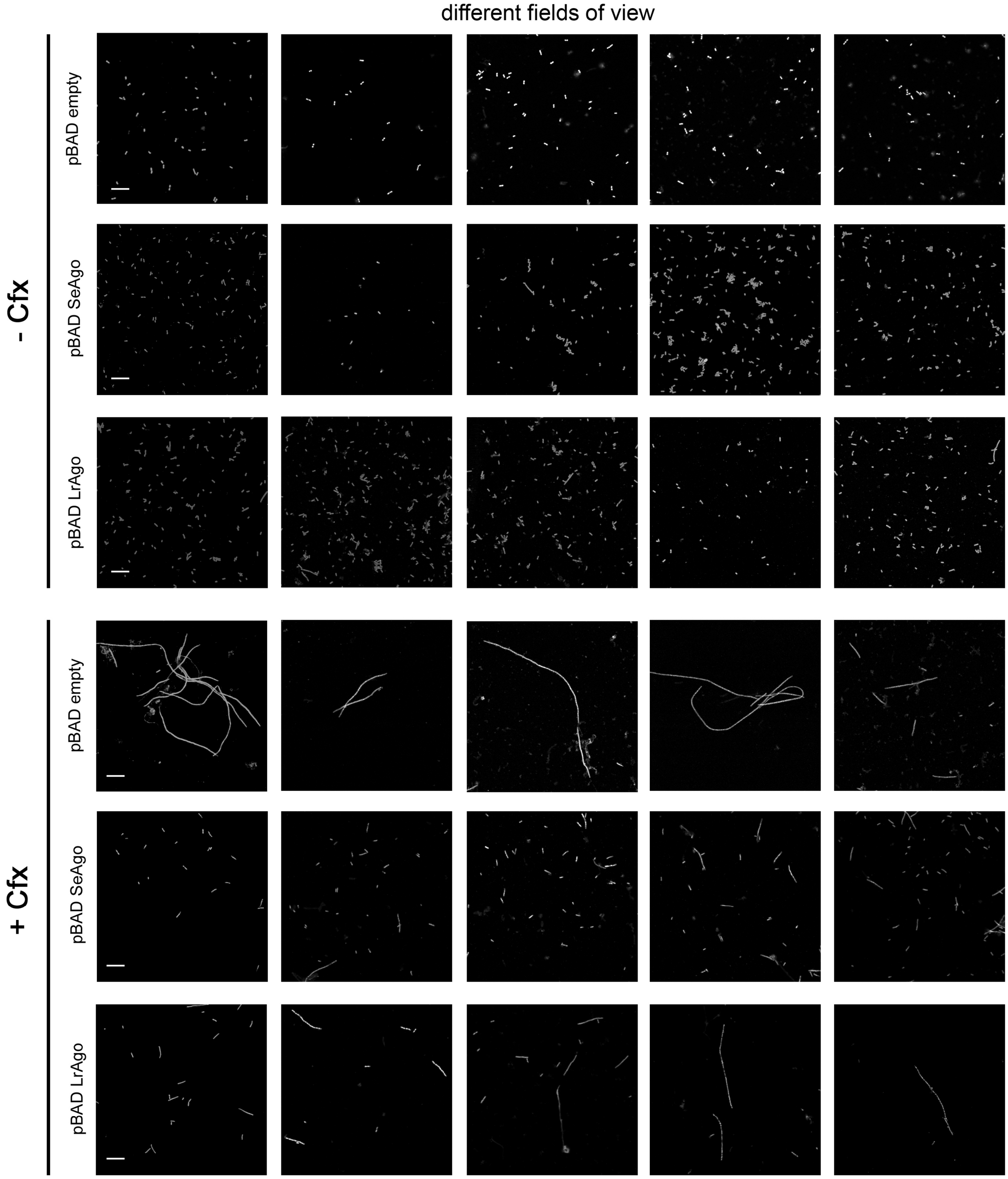

Fig. S3. Effects of pAgo expression on *E. coli* cell morphology. *E. coli* transformed with a control empty pBAD plasmid or pBAD ncoding pAgos were grown in the absence (top three rows) or in the presence (bottom three rows) of ciprofloxacin. The expression of pAgos was induced with 0.01% Ara. The samples were taken at 4.5 hours from the cultures shown in Fig. 3A. Fluorescence microscopy after acridine orange staining. The scale bar is 10 μm. The first column corresponds to images presented in Fig. 4, the other four images in each row are additional microscopic fields of view for the same samples.

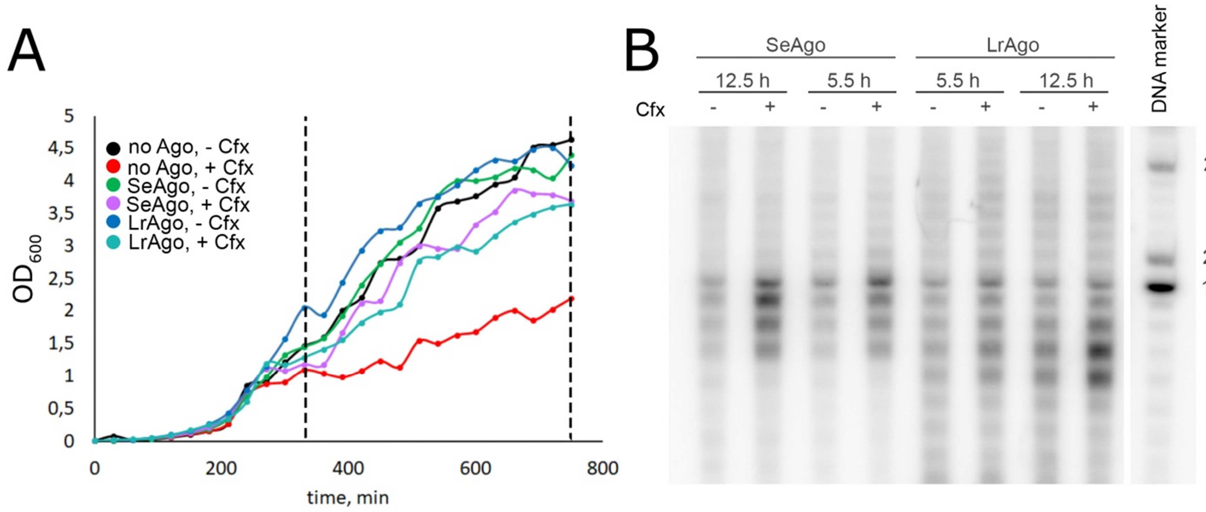

Fig. S4. Preparation of smDNA libraries from *E. coli* strains expressing pAgos. (A) Preparative-scale cultivation of *E. coli* strains lacking of containing pAgos. All cultures were grown in 0.5 liter of LB in the absence and in the presence of Cfx (0.3 ng/mL) and 0.01% Ara, and OD_{600} was monitored each 30 minutes. The dashed lines indicate 5.5 h and 12.5 h time points used for purification of pAgo-associated smDNAs. (B) Analysis of smDNAs purified from pAgos. SmDNAs isolated from pAgos after one-step purification (using Co^{2+}-affinity resin) were treated with alkaline phosphatase to remove pre-existing 5'-phosphates, labeled with γ-P^{32}-ATP and polynucleotide kinase and separated by 19% denaturing urea PAGE. The marker lane (M) contains 5'-labeled DNA oligonucleotides of indicated lengths.

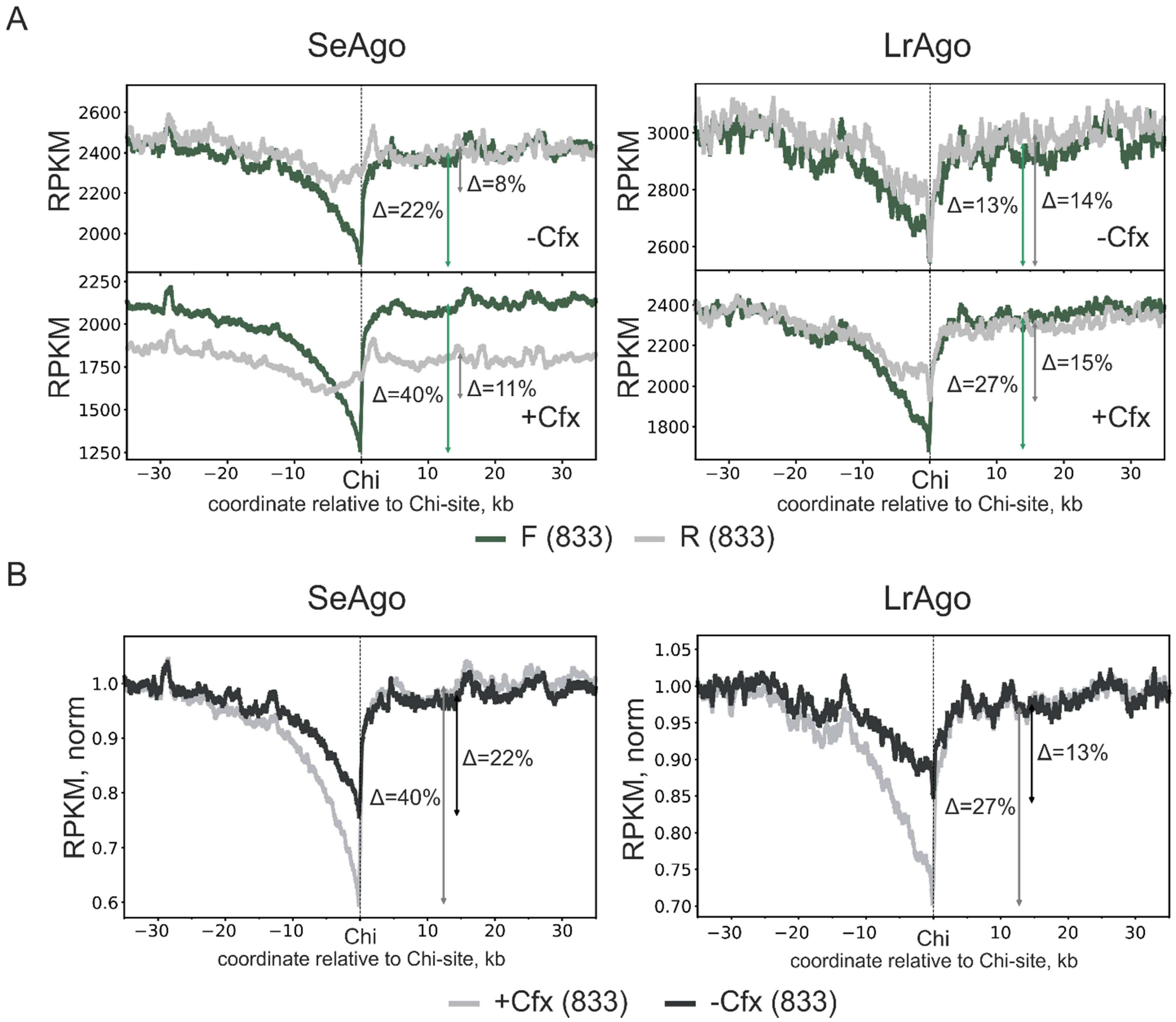

Fig. S5. Asymmetry of smDNA distribution around Chi sites at the stationary phase of growth. SmDNA analysis was performed as described in Fig. 7, but for the stationary cultures (12.5 hours of growth). Averages from two replicate experiments are shown. (A) Metaplots of the densities of smDNAs around Chi sites. SmDNA numbers were independently calculated for the DNA strands co-oriented (green, F) and oppositely oriented (gray, R) relative to the Chi sequence (5'-GCTGGTGG-3') for all Chi sites in both genomic strands and smoothened with a 400 bp sliding window. (B) Metaplots of the normalized densities of smDNAs around co-oriented Chi sites for *E. coli* strains grown in the absence and in the presence of Cfx (the data correspond to green smDNA profiles in panel A). Arrows indicate relative differences between the background and minimal densities of smDNAs around the Chi sites.

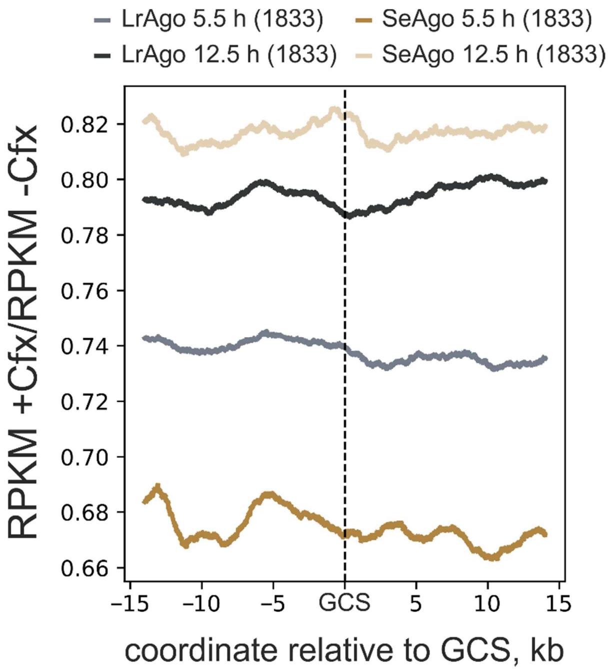

Fig. S6. Relative densities of pAgo-associated smDNAs around DNA gyrase cleavage sites (GCSs). Metaplots of the relative densities of smDNAs were calculated for 1833 GCSs identified in the reference genome (averages from two replicate experiments, calculated independently for the 5.5 h and 12.5 h time points). SmDNA densities were calculated for both DNA strands around GCSs for +Cfx and -Cfx conditions independently, and then the +Cfx density was divided by the -Cfx density. The resultant relative density was smoothened with a 2 kb sliding window.

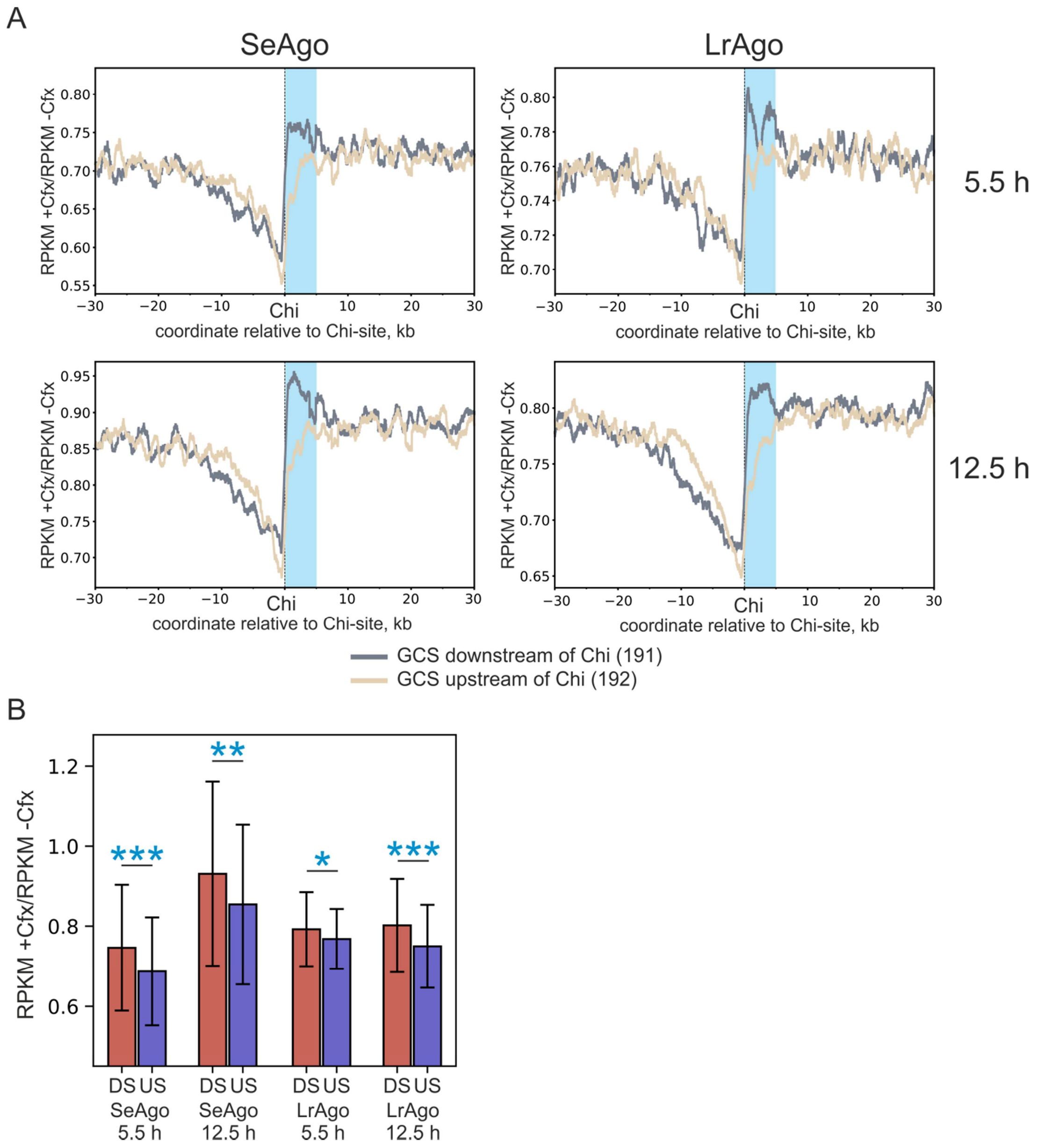

Fig. S7. Distribution of pAgo-associated smDNAs around Chi sites depending on the position of an adjacent GCS. (A) Metaplots of the relative densities of smDNAs around Chi sites adjacent to a downstream GCS (dark gray curves, 192 sites in total) and Chi sites adjacent to an upstream GCS (yellow curves, 191 sites in total) (averaged for two replicate experiments). A Chi site and a GCS were considered adjacent if there were no co-oriented Chi sites in between. SmDNA density was calculated for DNA strands co-oriented with Chi sites for the +Cfx and -Cfx conditions independently, and then the +Cfx density was divided by the -Cfx density. Resultant relative density was smoothened with a 1 kb sliding window. Blue rectangles mark the region (from the Chi sequence to +5 kb) used to quantify the relative enrichments of smDNAs in panel B. (B) Quantification of relative enrichments of smDNAs at the 3'-sides of Chi sites for Chi sites adjacent to a downstream GCS (DS, carmine) and Chi sites adjacent to an upstream GCS (US, violet). The error bars represent mean values ± STD for the same sets of Chi sites as in panel A. Enrichments were compared by a two-sided t-test. P-values of <0.05, <0.005 and <0.0005 are indicated with one, two, and three asterisks, respectively. The most significant differences in the smDNA enrichment are observed for SeAgo at the logarithmic phase of growth (5.5 h, p-value 1.1e-4) and for LrAgo at the stationary phase of growth (12.5 h, p-value 5.9e-6).

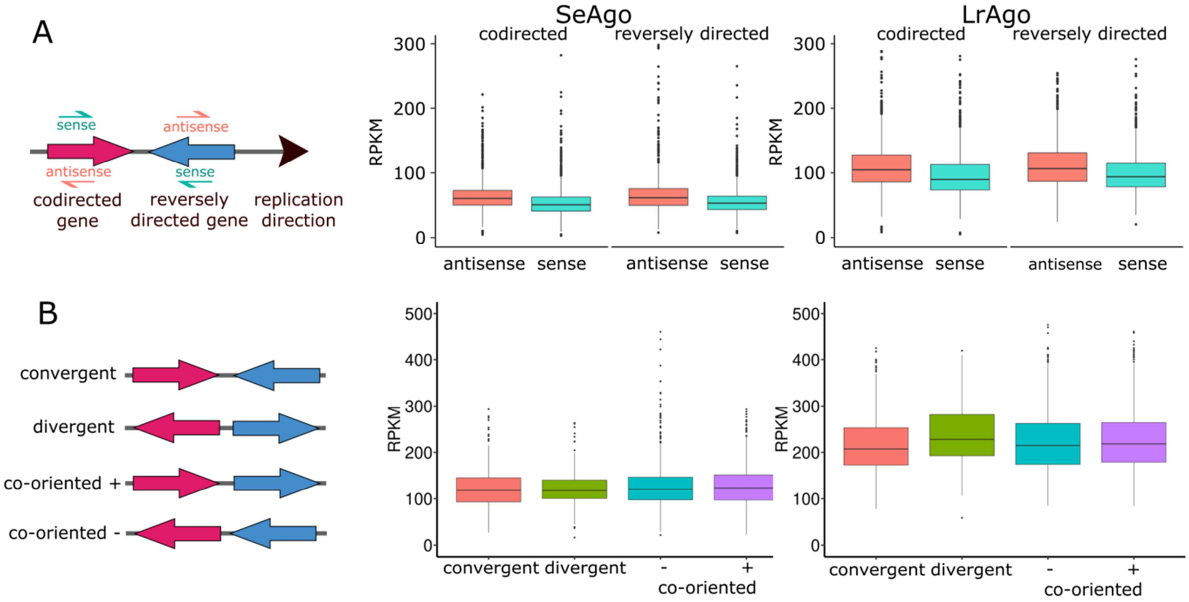

Fig. S8. Analysis of smDNA distribution relative to transcription units in *E. coli*. (A) Densities of smDNAs within genes co-directed or reversely directed relative to replication. For each gene, the amounts of smDNAs were calculated independently for the sense and antisense DNA strands, normalized by the gene length and expressed as RPKM (reads per kilobase per million aligned reads in the library). The data were independently averaged for each of the four types of orientation (sense and antisense strands for co-directed and reversely directed genes). (B) Densities of smDNAs in intergenic regions for convergent, divergent, and co-oriented gene pairs (located either in the plus or in the minus genomic strands). Only <500 bp intergenic regions were taken into account. SmDNA numbers were independently averaged for each of the four types of gene orientation (convergent, divergent, and two directions of co-oriented genes), normalized by the length of each intergenic region and expressed in RPKM. The data are shown as box plots (center line, median; box limits, upper and lower quartiles; whiskers, 1.5x interquartile range).

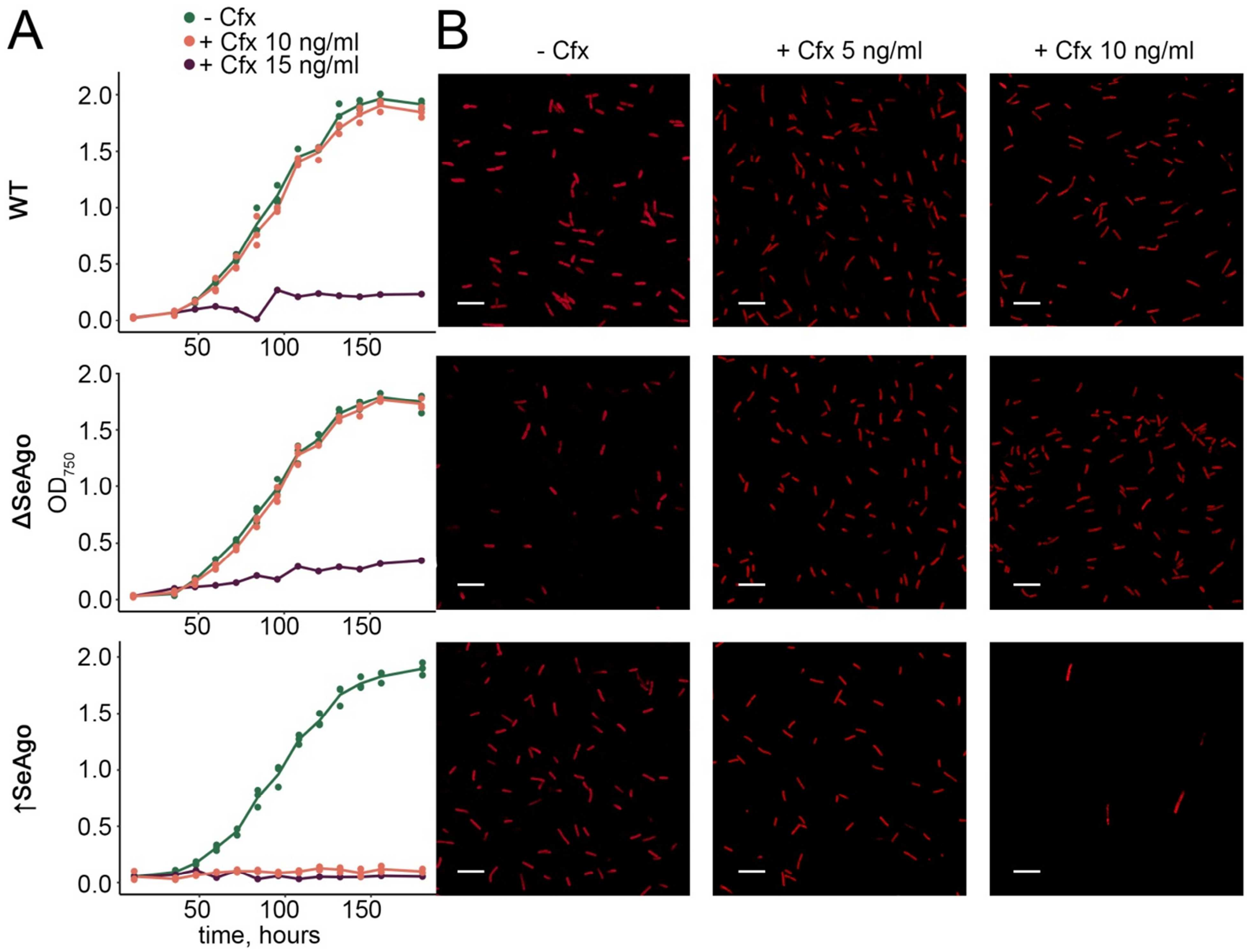

Fig. S9. No effect of SeAgo on cell growth in *S. elongatus***.** (A) Growth of *S. elongatus* strains with different levels of expression of SeAgo in the absence and in the presence of Cfx (10 or 15 ng/mL). Wt, wild-type strain; ΔSeAgo, strain without SeAgo; ↑SeAgo, strain with overexpression of SeAgo. Averages from 3 biological replicates are shown. (B) Analysis of cell morphology of the same *S. elongatus* strains in the absence or in the presence of Cfx (5 or 10 ng/mL). The samples were taken at 48 hours of growth and visualized by fluorescence microscopy. The scale bar is 10 μm.

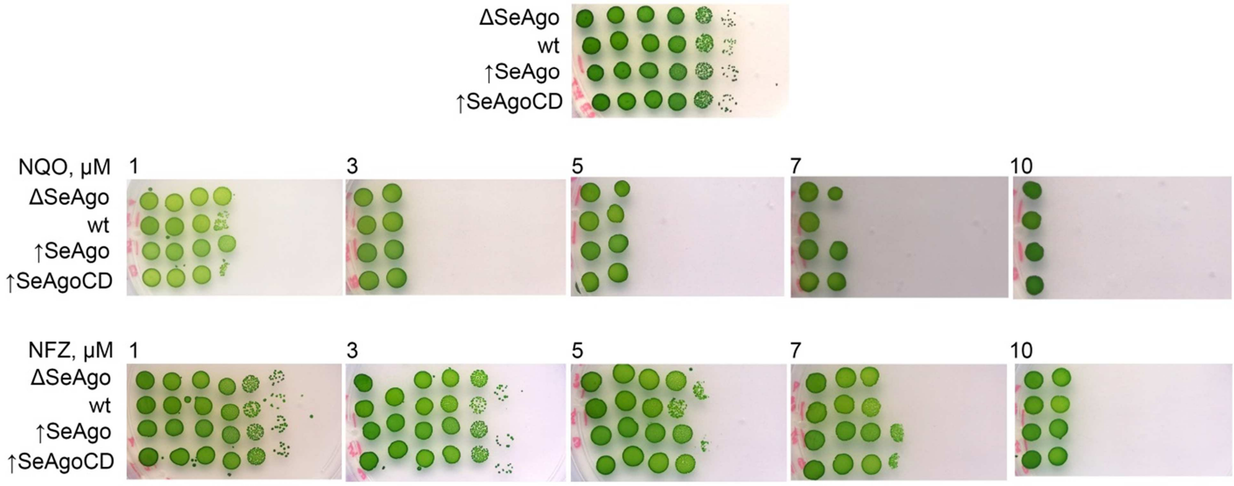

Fig. S10. Sensitivity of *S. elongatus* strains with various levels of expression of SeAgo to DNA damaging agents. (Top) Growth in the control conditions. (Middle) 4-NQO (4-nitroquinoline 1-oxide). (Bottom) NFZ (nitrofurazone). Wt, wild-type strain; ΔSeAgo, strain without SeAgo; ↑SeAgo, strain with overexpression of wild-type SeAgo; ↑SeAgoCD, strain with overexpression of a catalytically dead (CD) SeAgo with alanine substitutions of three aspartates in the catalytic tetrad (D516A, D584A and D709A) (Olina et al., 2020, ref. 28). Serial dilutions of each strain were plated on LB agar media containing increasing concentrations of 4-NQO and NFZ (from 1 to 10 μM) and grown for 12 days at 30 °C under constant light conditions (~250 µE $m^{-2}s^{-1}$).

Table S1. Small DNA libraries obtained and analyzed in this study. The data are available from the SRA database, bioproject number PRJNA878808

library number	library name	pAgo	ciprofloxacin	time, h	SRA accession	reads mapped to genome	reads mapped to genome, %	reads mapped to plasmid	reads mapped to plasmid, %	enrichment in plasmid coverage*
1	Se_5.5_nocip_rep_1	SeAgo	-	5.5	SRR21504308	368873	81.6	83301	18.4	11.4
2	Se_5.5_nocip_rep_2	SeAgo	-	5.5	SRR21508618	488441	79.3	127566	20.7	12.8
3	Se_5.5_cip_rep_1	SeAgo	0.3 μg/mL	5.5	SRR21508717	1509243	88.4	197824	11.6	7.2
4	Se_5.5_cip_rep_2	SeAgo	0.3 μg/mL	5.5	SRR21508866	1038304	87.2	152951	12.8	8
5	Se_12.5_nocip_rep_1	SeAgo	-	12.5	SRR21508879	791799	78.9	211831	21.1	13.1
6	Se_12.5_nocip_rep_2	SeAgo	-	12.5	SRR21508723	675406	77.7	193981	22.3	13.8
7	Se_12.5_cip_rep_1	SeAgo	0.3 μg/mL	12.5	SRR21508709	2152542	87.1	318401	12.9	8
8	Se_12.5_cip_rep_2	SeAgo	0.3 μg/mL	12.5	SRR21508927	2920406	86.4	460114	13.6	8.4
9	Lr_5.5_nocip_rep_1	LrAgo	-	5.5	SRR21508877	1266921	94.3	75910	5.7	3.5
10	Lr_5.5_nocip_rep_2	LrAgo	-	5.5	SRR21508707	1984106	94.8	108631	5.2	3.2
11	Lr_5.5_cip_rep_1	LrAgo	0.3 μg/mL	5.5	SRR21508916	1290785	92.5	105237	7.5	4.7
12	Lr_5.5_cip_rep_2	LrAgo	0.3 μg/mL	5.5	SRR21508919	1265045	91.3	121100	8.7	5.4
13	Lr_12.5_nocip_rep_1	LrAgo	-	12.5	SRR21508925	1196750	86.0	194585	14.0	8.6
14	Lr_12.5_nocip_rep_2	LrAgo	-	12.5	SRR21508926	1003480	87.4	145080	12.6	7.8
15	Lr_12.5_cip_rep_1	LrAgo	0.3 μg/mL	12.5	SRR21508713	2974923	83.1	606222	16.9	10.4
16	Lr_12.5_cip_rep_2	LrAgo	0.3 μg/mL	12.5	SRR21508921	1556966	84.5	285548	15.5	9.6

* The enrichment of plasmid-derived smDNAs is calculated as a ratio of the observed number of plasmid-mapped reads to the expected number of plasmid mapped reads. The expected number of reads = (genome-mapped reads + plasmid-mapped reads)×(plasmid size × copy number)/(genome size + plasmid size × copy number)

Table S2. Coordinates of genomic regions excluded from the meta-region analyses.
All coordinates are given for the NC_012971.2 reference genome.

Region	Coordinate start, bp	Coordinate end, bp	Comment
Del_0	19800	24620	Deletion
Del_1	267400	342300	Deletion
Del_2	412050	414460	Deletion
Del_3	543150	552050	Deletion
Del_4	646960	649240	Deletion
Del_5	746800	753100	Deletion
Del_6	759480	792100	Deletion
Del_7	871900	892700	Deletion
Del_8	1545400	1548500	Deletion
Del_9	1583600	1596750	Deletion
Del_10	1972880	1974400	Deletion
Del_11	1997050	2010200	Deletion
Del_12	2083200	2086730	Deletion
Del_13	2275700	2277200	Deletion
Del_14	2336400	2338980	Deletion
Del_15	2622360	2628500	Deletion
Del_16	2766840	2773900	Deletion
Del_17	2941200	2972380	Deletion
Del_18	2990385	2998800	Deletion
Del_19	3147270	3149570	Deletion
Del_20	3424090	3426940	Deletion
Del_21	3613050	3617500	Deletion
Del_22	3640150	3646350	Deletion
Del_23	3664850	3675250	Deletion
Del_24	3703780	3724700	Deletion
Del_25	3890420	3891465	Deletion
Del_26	3893500	3895130	Deletion

Del_27	3908170	3924150	Deletion
Del_28	3999410	4001330	Deletion
Del_29	4110090	4110560	Deletion
Del_30	4333100	4341060	Deletion
Del_31	4418100	4422100	Deletion
Del_32	4480150	4482400	Deletion
Del_33	4486630	4488600	Deletion
Del_34	4498680	4510000	Deletion
Ter region	1200000	1700000	*Ter* region, a highly-enriched region
rRNA H	226290	231970	*rRNA H* operon, a highly-enriched region
rRNA G	2592640	2597950	*rRNA G* operon, a highly-enriched region
rRNA E	3284580	3290217	*rRNA E* operon, a highly-enriched region
rRNA C	3831763	3837307	*rRNA C* operon, a highly-enriched region
rRNA A	3942020	3947620	*rRNA A* operon, a highly-enriched region
rRNA B	4074751	4079975	*rRNA B* operon, a highly-enriched region
rRNA D	4115870	4121450	*rRNA D* operon, a highly-enriched region
lacI	335300	337200	*lacI* gene, a highly-enriched region
rhsD	493000	496000	*rhsD* gene, a highly-enriched region
RS04425	878780	878960	*RS04425* orf, a highly-enriched region
RS25390	3822201	3823800	*RS25390* orf, a highly-enriched region
RS05470	1098501	1099800	*RS05470* orf, a highly-enriched region
